# Supplementary material for: Severe Neuro-COVID is associated with peripheral immune signatures, autoimmunity and neurodegeneration: a prospective cross-sectional study
Source: Nat Commun. 2022 Nov 9;13:6777. doi: 10.1038/s41467-022-34068-0 (PMC9645766; doi:10.1038/s41467-022-34068-0)
Supplement: Supplementary file 1 — Supplementary Information [file 41467_2022_34068_MOESM1_ESM.pdf]

Supplementary information

Severe Neuro-COVID is associated with peripheral immune signatures, autoimmunity and neurodegeneration: a prospective cross-sectional study

Etter et al.

Supplementary Figures

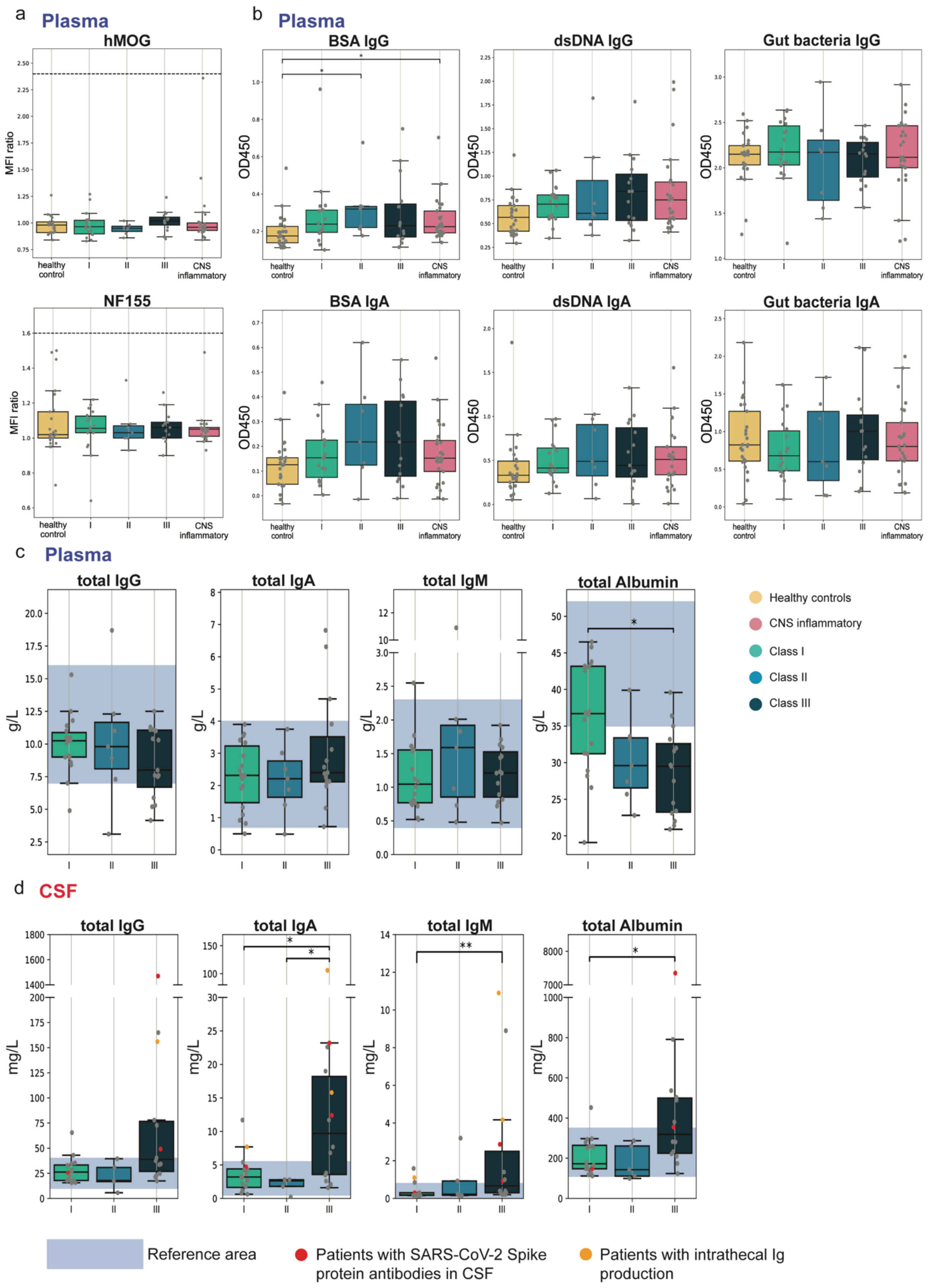

Supplementary Figure 1

Supplementary Figure 1: Antibody reactivities against known CNS myelin antigens and total Ig and albumin values per Neuro-COVID class.

Box plot representations (centre line at the median, upper bound at 75th percentile, lower bound at 25th percentile) of anti-(non)-self reactivities in plasma, total Ig and albumin levels in plasma (n=35) and cerebrospinal fluid (CSF) (n=40) of Neuro-COVID patients (n=40), and anti-myelin plasma reactivities (n=40) with whiskers at minimum and maximum values. Each dot represents one participant. **a** Box plot representation of plasma IgG reactivities against human myelin oligodendrocyte glycoprotein (hMOG) and neurofascin-155 (NF155), plotted as geometric median channel fluorescence (MFI) ratio. Vertical dotted line indicates the cut-off (hMOG: MFI ratio 2.4; NF155: MFI ratio 1.6) **b** Box plot representation of anti-BSA, -dsDNA, anti-gut bacteria (RePOOPulate)-IgG/IgA reactivities (OD_{450}; optical density at 450 nm) in plasma. **c** Box plot representation of total plasma IgG/A/M and albumin levels (g/L). Blue boxes indicate the clinical reference area. **d** Box plot representation of total CSF IgG/A/M and albumin levels (mg/L). Blue boxes indicate the clinical reference area.

Statistics (a-d): statistical significance was calculated using two-sided Mann-Whitney-U test and p-values were adjusted using Benjamin-Hochberg (BH)-procedure (adj. p: *<0.05, **<0.01, ***<0.001, if not otherwise indicated: not significant). Source data of a and b are provided as a Source Data file.

Legend: MFI: mean fluorescence intensity.

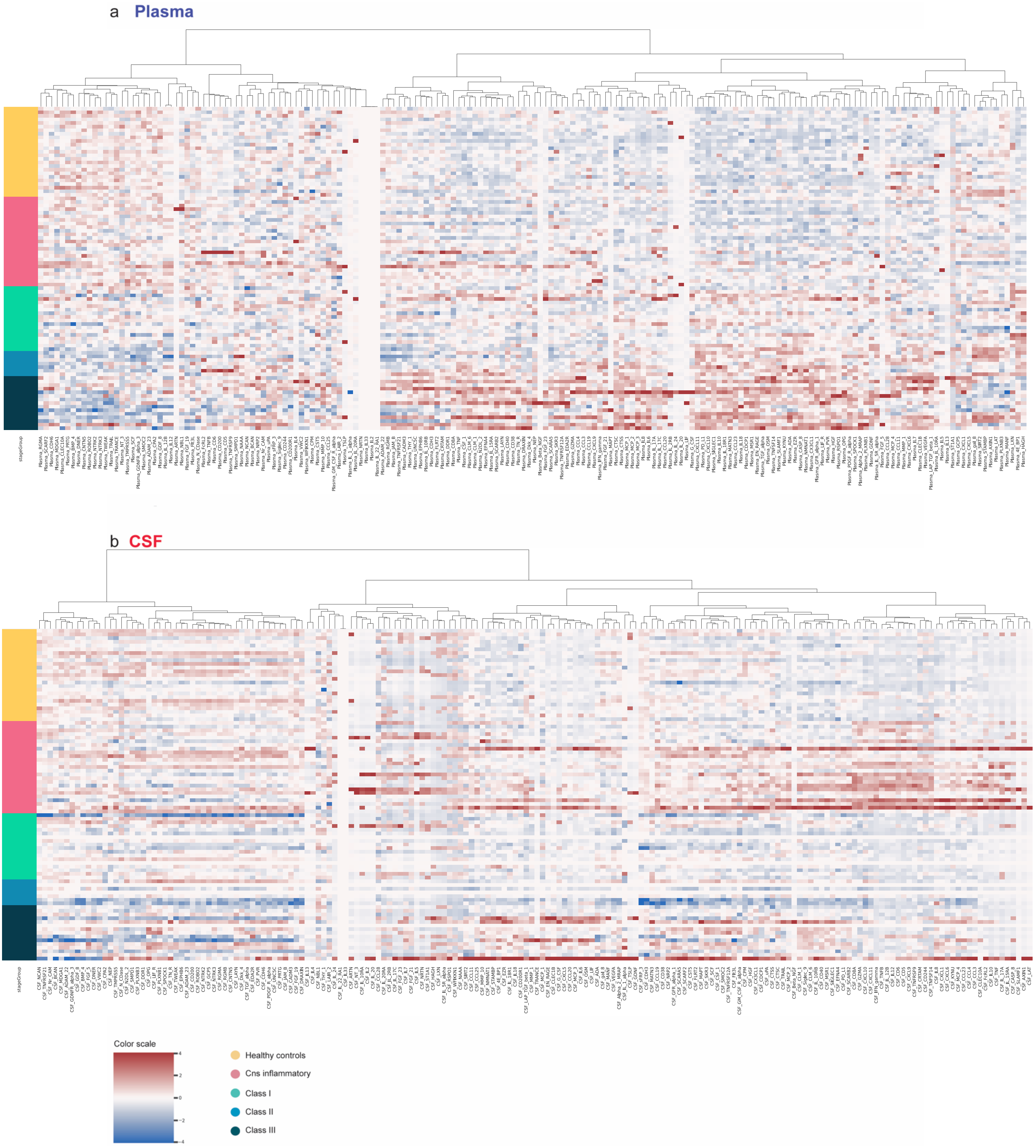

Supplementary Figure 2

Supplementary Figure 2: Heatmap of individual CSF and plasma analytes.

Z score clustered heatmap visualization for each Neuro-COVID patient (n=40), group (class I: n=18; class II: n=7; class III: n=15; CNS inflammatory controls: n=25; healthy controls: n=25) and sample source (CSF and plasma). **a** Plasma analytes are represented on the *x* axis. Each quadrant represents a single protein and individual participant. Participants and groups are represented on the *y* axis. *Z* scores are color-coded with red colors *(high)*, white *(neutral)* and blue colors *(low).* **b** Cerebrospinal fluid (CSF) analytes are represented on the *x* axis. Each quadrant represents a single protein and individual participant. Participants and groups are represented on the *y* axis. *Z* scores are color-coded with red colors *(high)*, white *(neutral)* and blue colors *(low)*.

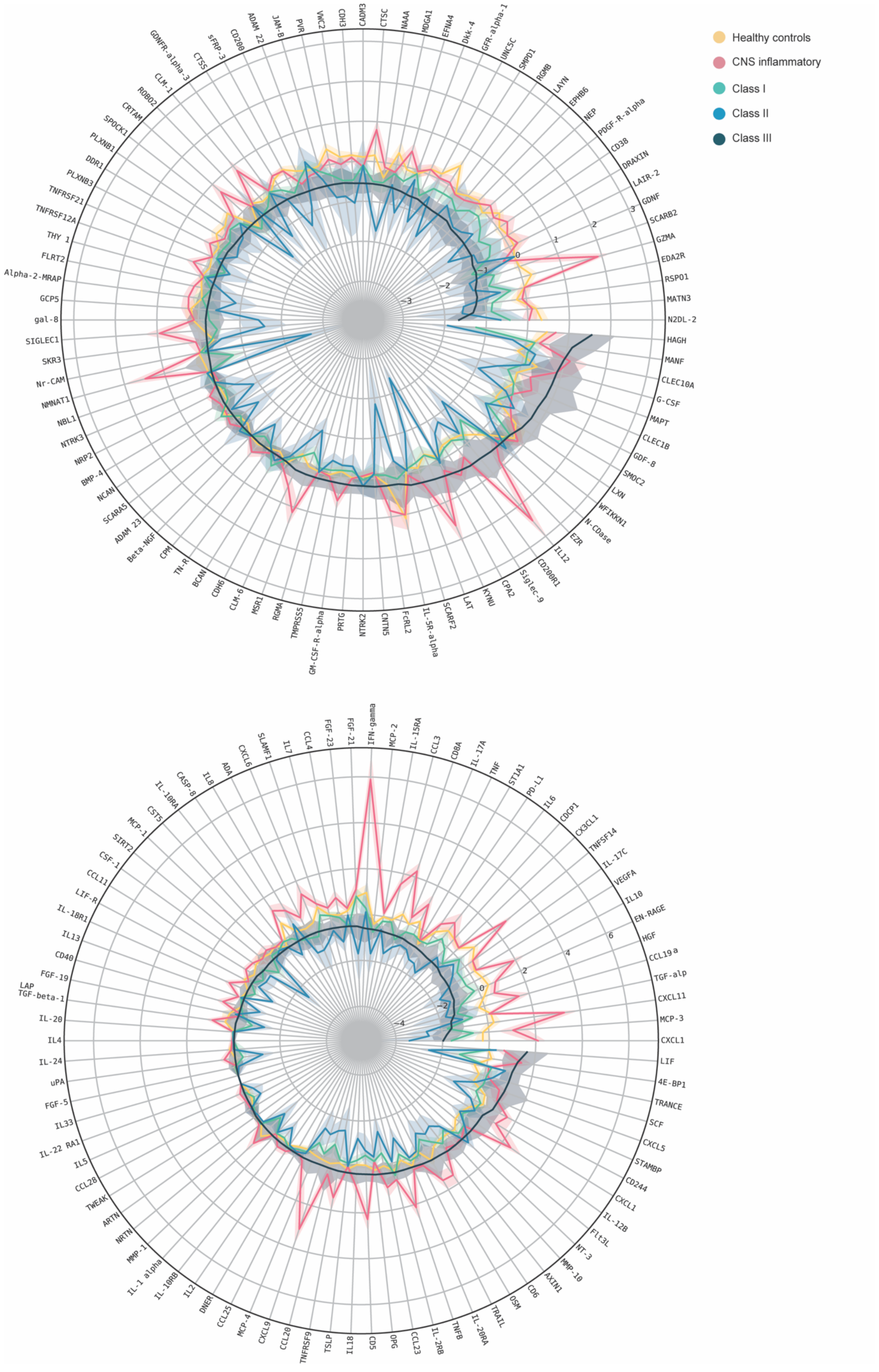

Supplementary Figure 3

Supplementary Figure 3: Low CSF/plasma soluble protein ratios are prevalent in Neuro-COVID patients indicating peripheral synthesis, whereas inflammatory controls display predominant intrathecal changes (rose plot presentation).

Rose plots illustrating the log_2-fold change of the cerebrospinal fluid (CSF)/plasma ratio for each cytokine in each group. A value of 0 corresponds to equality, a value of -1 refers to a 2 times less CSF concentration, a value of 1 means a 2 times less plasma concentration. For better visualization, the analytes were split into two separate rose plots. Range: standard error of mean (SEM). Source data are provided as a Source Data file.

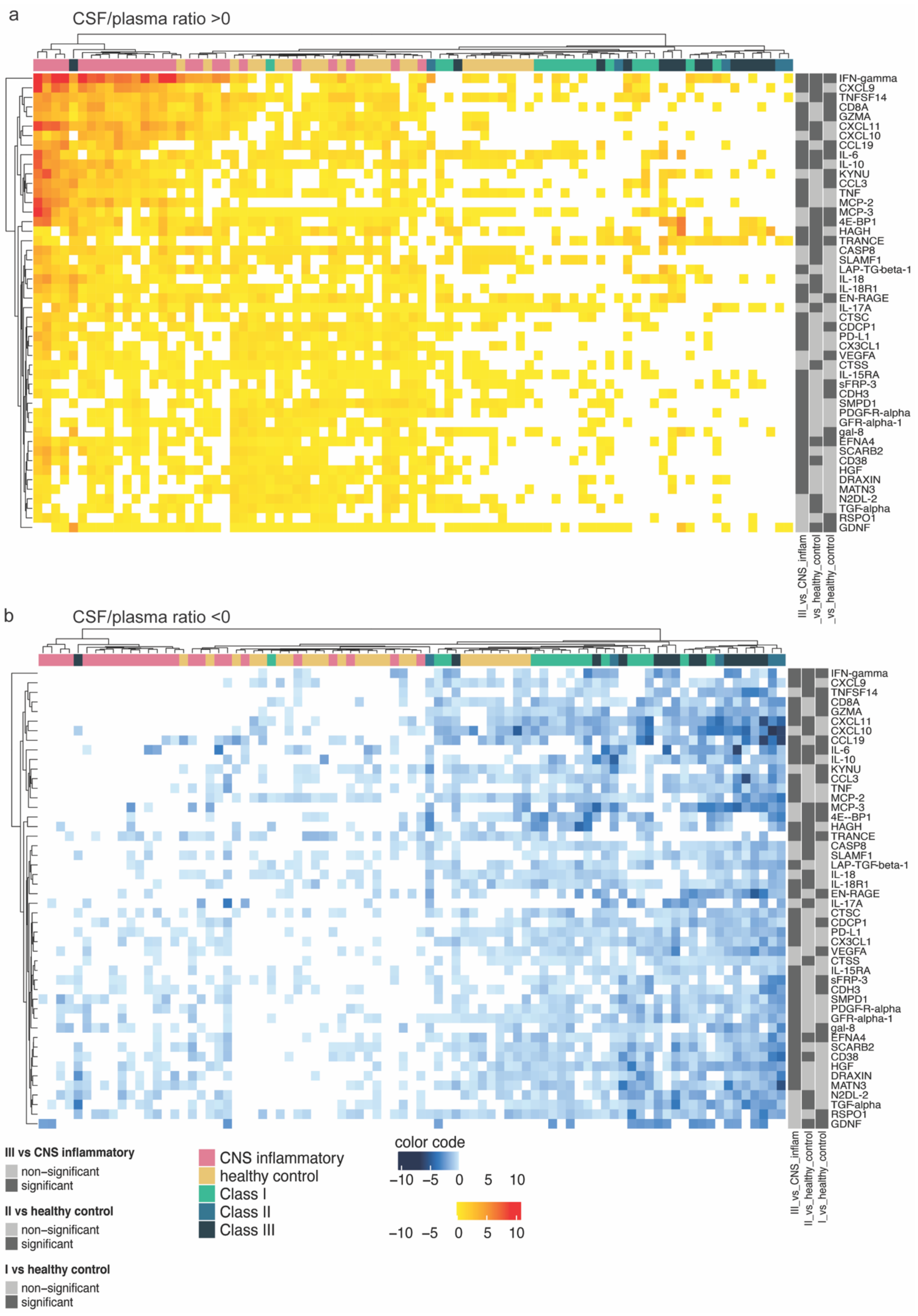

Supplementary Figure 4

Supplementary Figure 4: Low CSF/plasma soluble protein ratios are prevalent in Neuro-COVID patients indicating peripheral synthesis, whereas inflammatory controls display predominant intrathecal changes (heat map presentation).

Cerebrospinal fluid (CSF)/plasma ratios of each molecule were assessed to identify significant differences across groups. The ratio of 49 molecules significantly differed between groups after Benjamin-Hochberg (BH)-procedure. These molecules are represented on the *y* axis on the right of the heatmaps. Ratio values are color-coded with yellow-red colors illustrating a higher ratio *CSF/plasma ratio >0* and blue colors illustrating a lower ratio *CSF/plasma ratio <0*. The 3 vertical differently grey colored lines on the *y* axis demonstrate specific ratio differences between *(from left to right)* class III vs CNS inflammatory, class II vs healthy, and class I vs healthy controls. Heatmaps are separated into molecules with a *CSF/plasma ratio >0 (upper heatmap)*, indicating intrathecal synthesis, and a *CSF/plasma ratio <0 (lower heatmap)*, indicating peripheral synthesis. The main ratio changes in Neuro-COVID patients (n=40) take place in the plasma, whereas central nervous system (CNS) inflammatory controls display a stronger intrathecal immune response *(1)* compared to Neuro-COVID patients and *(2)* compared to immune reactions in their plasma. Source data are provided as a Source Data file.

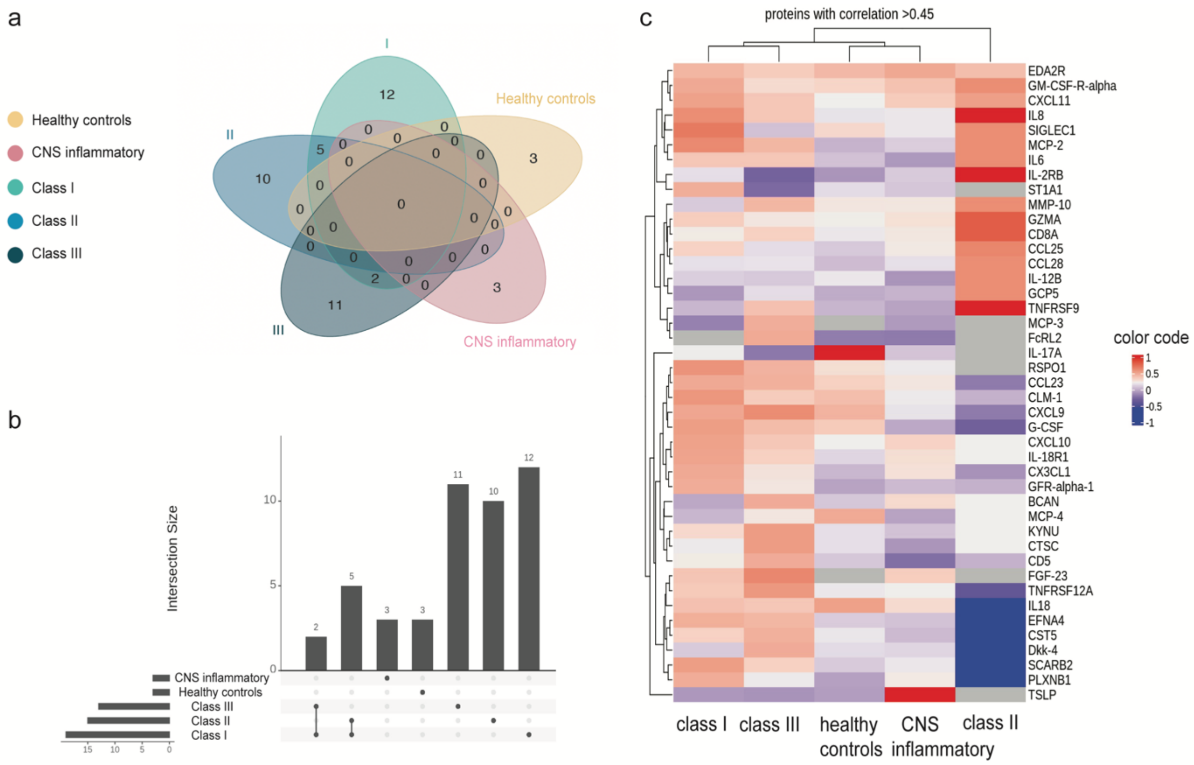

Supplementary Figure 5

Supplementary Figure 5: Individual CSF and plasma analytes with a strong CSF-plasma correlation.

Cerebrospinal fluid (CSF)-plasma correlation analysis with Venn diagram (a), UpSet plot (b) and a heatmap (c) of the strongest correlated genes (correlation coefficient >0.45).

a and **b** Venn diagram and UpSet plot demonstrate 10-12 class-defining proteins with a strong CSF-plasma correlation and only a few overlapping proteins. **c** CSF-plasma correlation values are color-coded, indicating strong correlation values in red color and low correlation values in blue color. Heatmap depicts a myeloid/eosinophil proinflammatory signature in class I patients, changing to a T-cell-mediated, proinflammatory feature in class II patients. Myeloid signature correlations are preserved in class II and partly overlap with class I patients. In class III, the strongest CSF-plasma correlation pattern is characterized by biomarkers implicating tissue damage and neuronal damage.

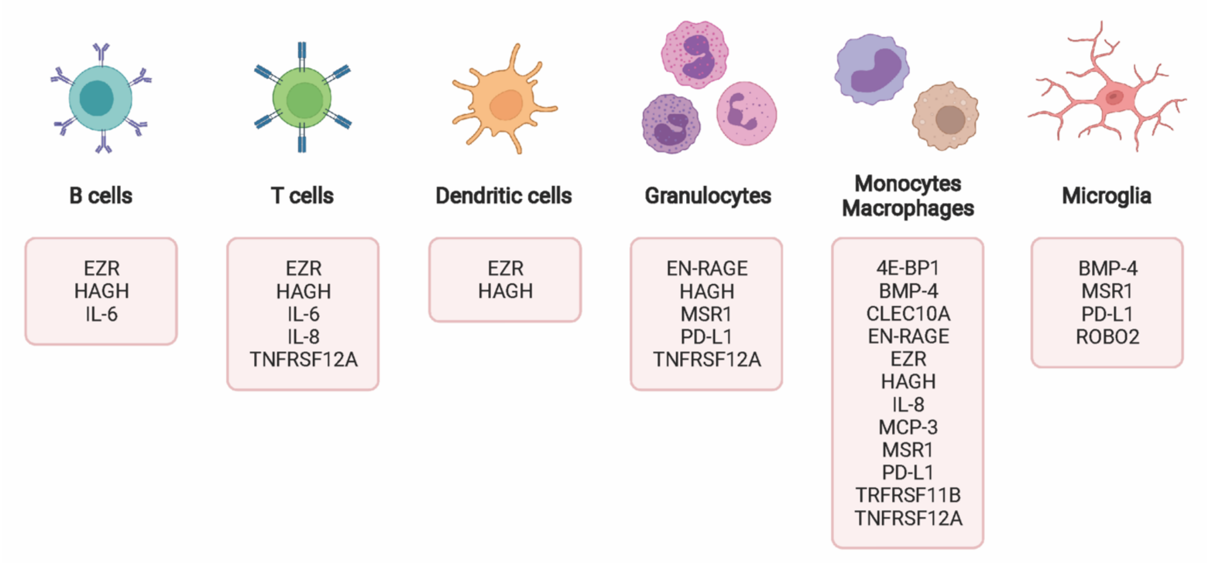

Supplementary Figure 6

Supplementary Figure 6: Immune cell sources of proteins associated with COVID-19 severity.

Microglia and innate immune cells like granulocytes, monocytes and macrophages are the sources of most cerebrospinal fluid (CSF) and plasma proteins associated with severe COVID-19 and Neuro-COVID development (4E-BP1, BMP-4, CLEC10A, EN-RAGE, EZR, HAGH, IL-8, MCP-3, MSR1, PD-L1, ROBO2, TRFRSF11B and TNFRSF12A). B and T lymphocytes and dendritic cells are the sources of a smaller number of proteins (EZR, HAGH, IL-6, IL-8, TNFRSF12A) with high predictive value for severe COVID-19 and Neuro-COVID class III development. The assessment of protein cell sources was based on mRNA expression in immune single cell types from a public scRNA-seq database. Data source: proteinatlas.org. Created with Biorender.com.

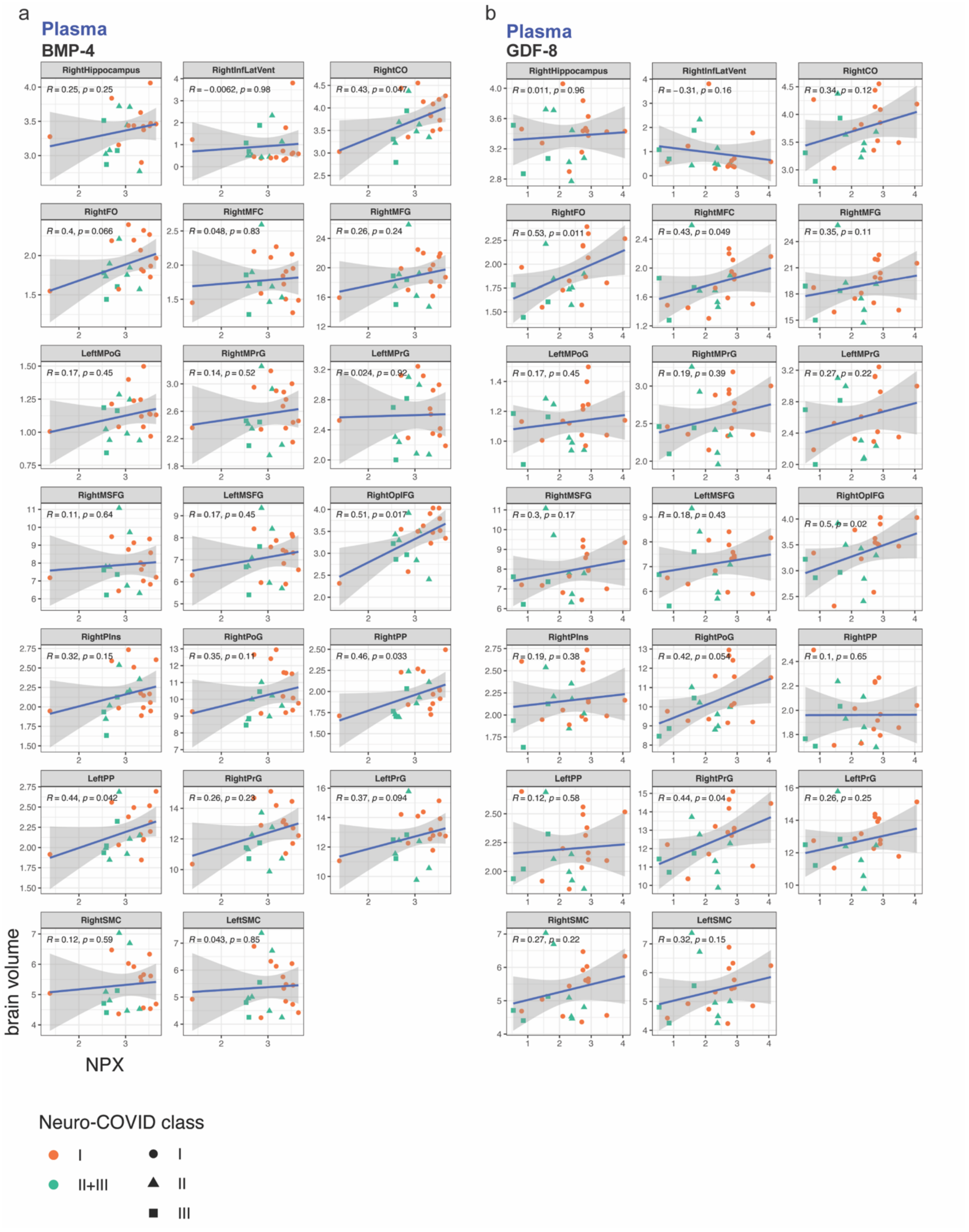

Supplementary Figure 7

Supplementary Figure 7: Elevated BMP-4 and GDF-8 plasma levels are associated with preserved GMVs in Neuro-COVID patients.

Spearman correlation scatter plots with linear regression (blue line) and the 95% confidence interval (gray band) demonstrating the correlation of **a** BMP-4 and **b** GDF-8 plasma levels and different regional brain volumes in Neuro-COVID patients (n=40). The *y* axis represents the regional brain volume values. The *x* axis represents the marginalized normalized protein expression (NPX) of the respective protein. Two-sided spearman correlation test was applied. None of the adjusted p-values was significant after Benjamin-Hochberg (BH)-procedure.

Legend: R: Spearman correlation coefficient, p: associated p-value, RightFO: right frontal operculum, LeftPrG: left precentral gyrus, RightOpIFG: right opercular part of the inferior frontal gyrus, RightInfLatVent: right inferior lateral ventricle, LeftPP: left planum polare, RightPIns: right posterior insula, RightCO: right central operculum.

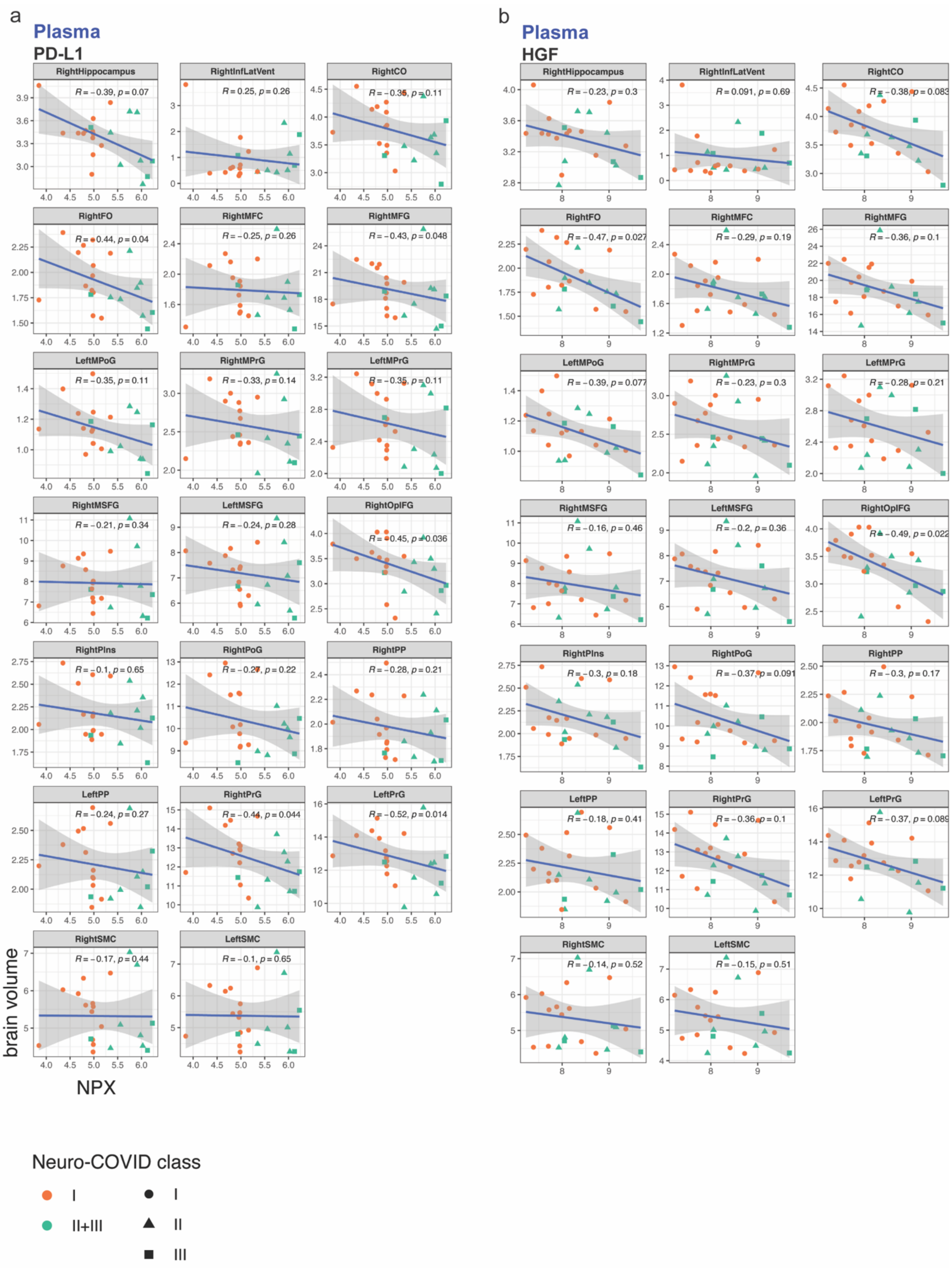

Supplementary Figure 8

Supplementary Figure 8: Elevated PD-L1 and HGF plasma levels are associated with decreased GMVs in Neuro-COVID patients.

Spearman correlation scatter plots with linear regression (blue line) and the 95% confidence interval (gray band) demonstrating the correlation of **a** PD-L1 and **b** HGF plasma levels and different regional brain volumes in Neuro-COVID patients (n=40). The *y* axis represents the regional brain volume values. The *x* axis represents the marginalized normalized protein expression (NPX) of the respective protein. Two-sided spearman correlation test was applied. None of the adjusted p-values was significant after Benjamin-Hochberg (BH)-procedure.

Legend: R: Spearman correlation coefficient, p: associated p-value, RightFO: right frontal operculum, LeftPrG: left precentral gyrus, RightOpIFG: right opercular part of the inferior frontal gyrus, RightInfLatVent: right inferior lateral ventricle, LeftPP: left planum polare, RightPIns: right posterior insula, RightCO: right central operculum.

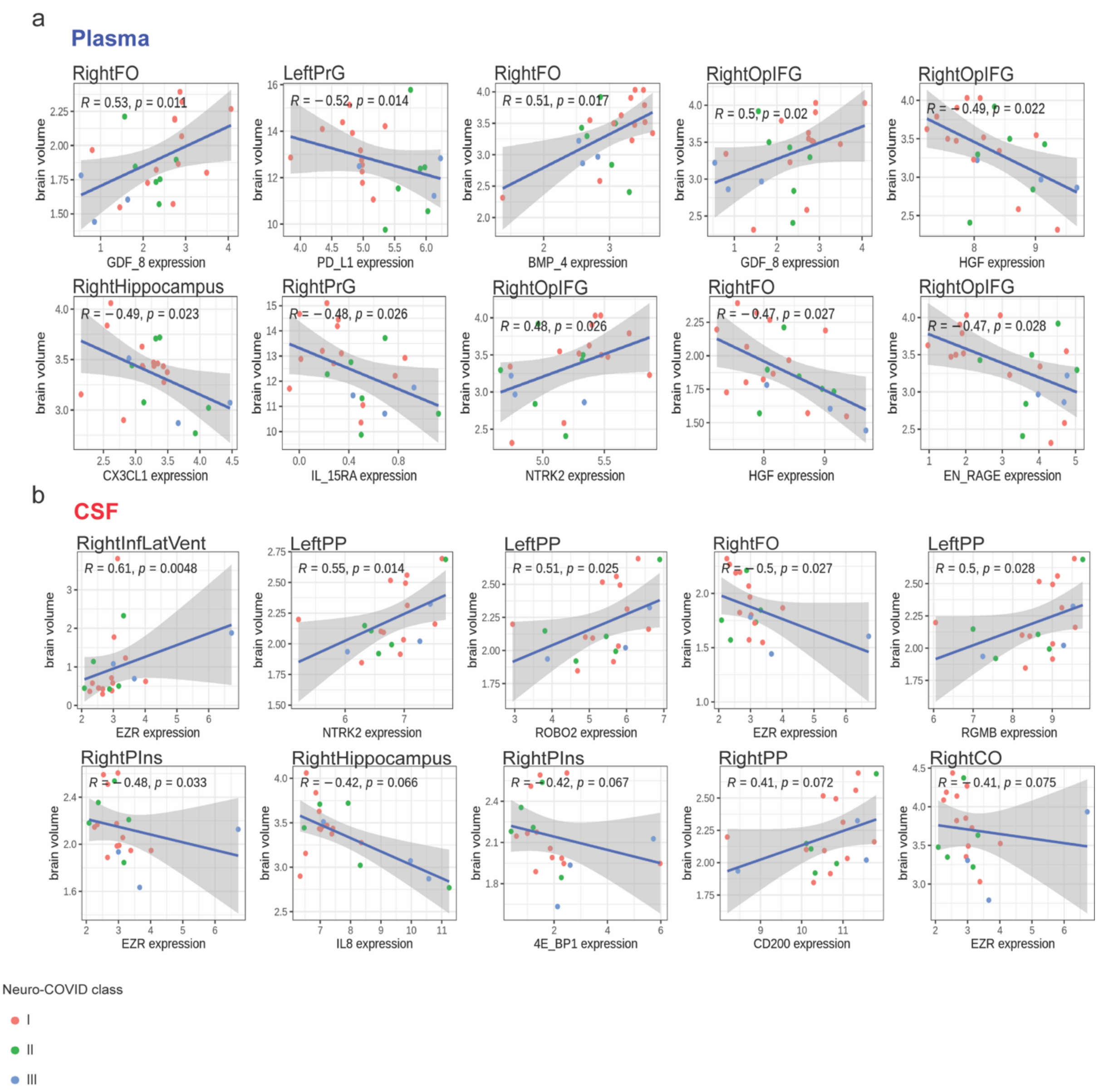

Supplementary Figure 9

Supplementary Figure 9: Correlation analysis of brain regions and proteins with most significant associations.

a B Spearman correlation scatter plots with linear regression (blue line) and the 95% confidence interval (gray band) of marginalized normal protein expression (NPX) of individual plasma analytes associated with *decreased regional brain volumes:* PD-L1, HGF, CX3CL1, IL-15RA, EN-RAGE; and plasma analytes associated with *protective effects on regional brain volumes:* GDF-8, BMP-4, NTRK2. Statistics: none of the p-values are significant after Benjamin-Hochberg (BH)-procedure. **b** Boxplot representations of marginalized NPX individual cerebrospinal fluid (CSF) analytes associated with *decreased regional brain volumes:* EZR, IL-8, 4E-BP1; and CSF analytes associated with *protective effects on regional brain volumes:* EZR, NTRK2, ROBO2, RGMB, CD200. Two-sided spearman correlation test was applied. None of the p-values are significant after BH-procedure.

Legend: R: Spearman correlation coefficient, p: associated p-value, RightFO: right frontal operculum, LeftPrG: left precentral gyrus, RightOpIFG: right opercular part of the inferior frontal gyrus, RightInfLatVent: right inferior lateral ventricle, LeftPP: left planum polare, RightPIns: right posterior insula, RightCO: right central operculum, R: correlation coefficient, p: p-value.

Supplementary Tables

**Healthy control cases** (n=25)	
**Age, years, mean (SD)**	52 (18)
Range, years	23-75
**Sex**	
Female, n (%)	12 (48%)
Male, n (%)	13 (52%)
**CNS inflammatory control cases** (n=25)	
**Age, years, mean (SD)**	54 (19)
Range, years	20-82
**Sex**	
Female, n (%)	12 (48%)
Male, n (%)	13 (52%)
**Neurologic disorder**	
Postherpetic neuralgia, n (%)	1 (4%)
Herpetic meningitis, n (%)	1 (4%)
Herpetic encephalitis, n (%)	1 (4%)
Herpetic meningoencephalitis, n (%)	1 (4%)
VZV meningomyeloradiculitis, n (%)	1 (4%)
Disseminated herpes zoster with CNS affection, n (%)	3 (12%)
Viral meningitis (not specified), n (%)	2 (8%)
Viral meningoencephalitis (not specified), n (%)	1 (4%)
Relapsing meningitis of unclear etiology, n (%)	1 (4%)
Eosinophilic encephalitis, n (%)	3 (12%)
Cranial neuropathy, n (%)	1 (4%)
Tuberculous meningoencephalitis, n (%)	3 (12%)
Neuroborreliosis, n (%)	3 (12%)
Red nucleus lesion of unclear etiology, n (%)	1 (4%)
Neurosarcoidosis, n (%)	1 (4%)
Susac's syndrome, n (%)	1 (4%)
Autoimmune encephalitis, n (%)	1 (4%)
Rasmussen encephalitis, n (%)	1 (4%)
SD: standard deviation<br>VZV: varicella-zoster virus<br>CNS: central nervous system	

Supplementary Table 1: Characteristics of patients with a non-MS inflammatory neurologic disorder and healthy controls.

Demographics of healthy individuals (n=25) and non-MS inflammatory neurologic disorder patients (CNS inflammatory controls, n=25). For non-MS inflammatory neurologic disorder patients, specific neurological conditions are depicted.

Legend: SD: standard deviation, VZV: varicella-zoster virus, CNS: central nervous system.

	Class I (n=18)	Class II (n=7)	Class III (n=15)
**CSF characteristics**			
**Protein levels**			
Missing, n	4	2	1
Protein levels, mg/L, mean (SD)	334 (124)	282 (114)	1424 (3056)
Range, mg/L	103-673	174-429	286-12000
Elevated protein (>500mg/L), n (%)	1 (7.1%)	0 (0%)	10 (71.4%)
**Leukocyte count**			
Missing, n	5	2	2
Leukocyte count, x10^6/L, mean (SD)	3 (3)	3 (2)	12 (27)
Range, x10^6/L	1-12	1-7	0-99
Elevated leukocyte count (≥5x10^6/L), n (%)	1 (7.7%)	1 (20%)	6 (46.2%)
**Albumin ratio (CSF/plasma)**			
Missing, n	6	4	6
Ratio, mean (SD)	5.2 (2.1)	5.2 (3.1)	13.1 (7.8)
Elevated ratio (≥8.2x10^{-3}), n (%)	1 (8.3%)	1 (33.3%)	6 (66.7%)
**Glucose levels**			
Missing, n	4	2	2
Glucose levels, mmol/L, mean (SD)	4.3 (1.2)	4.8 (1.5)	5.3 (1.8)
Range, mmol/L	3.2-7.6	3.5-7.2	2.9-7.9
Elevated glucose (>6.1mmol/L), n (%)	1 (7.1%)	1 (20%)	4 (30.8%)
**Glucose ratio (CSF/plasma)**			
Missing, n	9	3	1
Ratio, mean (SD)	0.6 (0.1)	0.5 (0.1)	0.7 (0.1)
**Lactate levels**			
Missing, n	4	2	3
Lactate levels, mmol/L, mean (SD)	1.7 (0.3)	2.0 (0.7)	2.8 (1.9)
Range, mmol/L	1.4-2.2	1.3-2.9	1.6-8.4
Elevated lactate (>2mmol/L), n (%)	3 (21.4%)	2 (40%)	9 (75%)

Supplementary Table 2: CSF characteristics of Neuro-COVID patients per class.

Detailed cerebrospinal fluid (CSF) characteristics of all Neuro-COVID patients donating CSF samples (n=35), including protein levels, the leukocyte count, CSF/plasma Albumin ratio, CSF glucose levels, the CSF/plasma glucose ratio and CSF lactate levels. Legend: SD: standard deviation

**Class I** (n=4)	
**Age, years, mean (SD)**	64 (10)
Range, years	51-80
**Sex**	
Female, n (%)	2 (50%)
Male, n (%)	2 (50%)
**Preexisting disorders**	
Chronic kidney disease, n (%)	1 (25%)
Arterial hypertension, n (%)	3 (75%)
Coronary heart disease, n (%)	1 (25%)
**Main neurological symptom at presentation**	
Headache	3 (75%)
Anosmia	1 (25%)
**Class III** (n=4)	
**Age, years, mean (SD)**	64 (13)
Range, years	48-78
**Sex**	
Female, n (%)	1 (25%)
Male, n (%)	3 (75%)
**Preexisting disorders**	
No past medical history, n (%)	1 (25%)
Arterial hypertension, n (%)	3 (75%)
Diabetes mellitus, n (%)	2 (50%)
Chronic obstructive pulmonary disease, n (%)	1 (25%)
Coronary heart disease, n (%)	1 (25%)
Cancer of any type, n (%)	1 (25%)
**Main neurological symptom at presentation**	
Encephalopathy, n (%)	4 (100%)

Supplementary Table 3: Patient characteristics of class I and class III Neuro-COVID patients selected for B cell receptor sequencing.

Demographics, past medical history and main neurological symptom at presentation of selected class I (n=4) and class III (n=4) Neuro-COVID patients.

Structures	Control (mean)	Control (sd)	Patient (mean)	Patient (sd)	t stat	pval uncor	pval corr
**R Hippocampus**	3.321	0.388	3.360	0.349	-2.227	**0.035**	0.297
**R Inf Lat Vent**	0.697	0.302	0.923	0.754	-2.132	**0.043**	0.297
**R Central Operculum**	4.005	0.430	3.693	0.471	2.137	**0.042**	0.297
**R Frontal Operculum**	2.027	0.264	1.843	0.260	2.288	**0.031**	0.297
**R Medial Frontal Cortex**	1.889	0.234	1.780	0.290	2.222	**0.036**	0.297
**R Middle Frontal Gyrus**	18.939	2.250	18.425	2.590	2.062	**0.049**	0.315
**L Medial Postcentral Gyrus**	1.184	0.180	1.102	0.159	2.313	**0.029**	0.297
**R Medial Precentral Gyrus**	2.667	0.355	2.510	0.347	2.213	**0.036**	0.297
**L Medial Precentral Gyrus**	2.714	0.371	2.524	0.375	3.237	**0.003**	0.190
**R Medial Superior Frontal Gyrus**	8.277	1.092	7.843	1.165	2.659	**0.013**	0.275
**L Medial Superior Frontal Gyrus**	7.376	0.873	7.006	0.980	2.246	**0.034**	0.297
**R Posterior Insula**	2.371	0.292	2.164	0.285	2.362	**0.026**	0.297
**R Postcentral Gyrus**	10.984	1.546	10.299	1.287	2.398	**0.024**	0.297
**R Planum Polare**	2.101	0.238	1.946	0.228	2.331	**0.028**	0.297
**L Planum Polare**	2.327	0.244	2.178	0.259	2.150	**0.041**	0.297
**R Precentral Gyrus**	12.895	1.575	12.222	1.515	2.617	**0.015**	0.275
**L Precentral Gyrus**	13.296	1.589	12.590	1.454	3.238	**0.003**	0.190
**R Supplementary Motor Cortex**	5.461	0.713	5.243	0.712	2.698	**0.012**	0.275
**L Supplementary Motor Cortex**	5.569	0.733	5.243	0.806	2.914	**0.007**	0.228

Supplementary Table 4: Smaller regional brain volumes in Neuro-COVID patients compared to the volumetric imaging control group.

Twenty brain regions were constituted with a lesser volume in the Neuro-COVID group (n=35) compared to healthy controls (n=36). The regional volumes were compared between groups using a linear regression model. Additional covariates were age, sex, age*sex interaction, MRI magnetic field strength and total intracranial volume (TIV). One-sided Levene's test of equal variances was used to assess whether dependent variable's variance is equal in both groups. Statistics: there were no significant

differences after false discovery rate (FDR) correction. Significant correlation values, $p < 0.05$ (uncorrected), are represented in bold.

Legend: sd: standard deviation, uncorr: uncorrected, corr: corrected, R: right, L: left, Inf: inferior, lat: lateral, Vent: ventricle.

	COVID-19 patients	Controls
**Numbers of subjects**	35	36
**Age**	51.9, sd (19.7)	54.03, sd (23.7)
**Sex, Male/Female**	M/F (14/21)	(13/23)
**stroke**	2	na
**CSF Leukocytes**	17 (4.29, sd 3.72)	na
**CSF Lactate**	18 (1.89, sd 0.50)	na
**CSF Protein**	n=18 (346.11, sd 180.83)	na
**CSF Blood Albumin Ratio**	n=15 (6.50, sd 4.60)	na
**CSF Glucose**	n=18 (4.49, sd 1.30)	na
**Plasma TRANCE**	n=20 (2.74, sd 0.93)	na
**Plasma EN-RAGE**	n=20 (3.30, sd 1.37)	na
**CSF OPG**	n=18 (9.55, sd 0.68)	na
**CSF TRANCE**	n=18 (-0.31, sd 0.29)	na
**CSF EN-RAGE**	n=18 (0.43, sd: 0.74)	na
**Died in Hospital**	1	na
**Neurocovid state**	n=1 (13), 2 (6), 3 (2)	na
**Weight [kg]**	69.88 (sd 11.95)	71.53(sd 16.30)
**Height [meters]**	1.69 (sd 0.09)	1.69 (sd 0.11)
**MRI magnetic Field strength**	1.5 T (30)<br>3 T (5)	1.5 T (11)<br>3 T (25)
**TIV [cm^3]**	1431(sd 141,96)	1497(sd 156.293)
**Global gray matter [cm^3]**	618.65 (sd 98.86)	638.61 (sd 95.38)
**Global White matter [cm^3]***	449.28 (sd 63.76)	451.09 (sd 62.63)
**CSF matter [cm^3]**	373.04 (sd 137.65)	408.01 (sd 147.46)
**BPF**	0.74 (sd 0.08)	0.73 (sd 0.08)
**Parenchyma Volume [cm^3]**	1067.94 (sd 138.22)	1089.70 (sd 137.40)

Supplementary Table 5: Regional brain volume values for the volumetric imaging Neuro-COVID group and control group.

Brain regional volume differences between Neuro-COVID patients (n=35) and the control group (n=36). Normal distributions of all variables were assessed using Shapiro-Wilk test and visual inspection of the histograms. To test the variances' equality, Levene's test was applied. Clinical and demographic variables were compared between groups with t-test for independent groups, Mann-Whitney-U test or Chi-square test where appropriate. All applied tests were one-sided. Source data are provided as a Source Data file.

Legend: sd: standard deviation, uncorr: uncorrected, corr: corrected. Significant correlations values, $p<0.05$ (uncorrected), are represented in bold.

Structures	CSF Leukocytes corr	pval	CSF Protein corr	pval	CSF Blood Albumin Ratio corr	pval
**L Accumbens Area**	-0.821	**0.029**	-0.709	0.054	-0.767	0.059
**LAmygdala**	-0.687	0.081	-0.744	**0.049**	-0.740	0.078
**L Basal Forebrain**	-0.749	0.050	-0.664	0.067	-0.657	0.111
**L Fusiform Gyrus**	-0.721	0.060	-0.788	**0.036**	-0.750	0.073
**L Inferior Temporal Gyrus**	-0.761	0.050	-0.599	0.104	-0.704	0.082
**L Medial Orbital Gyrus**	-0.748	0.050	-0.601	0.104	-0.687	0.089
**L Posterior Cingulate Gyrus**	-0.746	0.050	-0.593	0.105	-0.528	0.266
**Optic Chiasm**	-0.639	0.123	-0.758	**0.044**	-0.829	**0.028**
**R Anterior Orbital Gyrus**	-0.805	**0.029**	-0.763	**0.044**	-0.806	**0.040**
**R Amygdala**	-0.727	0.058	-0.832	**0.015**	-0.838	**0.028**
**R Angular Gyrus**	-0.432	0.329	-0.688	0.062	-0.837	**0.028**
**R Entorhinal Area**	-0.849	**0.029**	-0.866	**0.008**	-0.899	**0.009**
**R Lateral Orbital Gyrus**	-0.816	**0.029**	-0.632	0.078	-0.639	0.118
**R Posterior Orbital Gyrus**	-0.795	**0.031**	-0.714	0.054	-0.793	**0.046**
**R Putamen**	-0.588	0.157	-0.731	0.049	-0.786	**0.046**
**R Superior Frontal Gyrus**	-0.571	0.172	-0.733	0.049	-0.705	0.082

Supplementary Table 6: Significant associations of regional brain volumes and clinical variables.

The significant associations between regional brain volume and clinical variables in the Neuro-COVID group (n=35) were assessed using partial correlation, allowing to calculate the linear partial correlation between our variables of interest adjusting for different covariates. Covariates were age, sex, age*sex interaction, MRI magnetic field strength and TIV. To adjust for multiple comparisons a false discovery rate (FDR) method was used. All correlation values were negative. For each region the partial correlation value and their corrected p-value is shown. Source data are provided as a Source Data file.

Legend: In bold, significant FDR p-values corrected ($p < 0.05$). CSF: Cerebrospinal fluid, L: left, R: right, pval: p values corrected by FDR, corr: correlation.

	Class I (n=18)	Class II (n=7)	Class III (n=15)
**13-months follow-up**			
Follow-up performed, n (%)	18 (100%)	7 (100%)	8 (53.3%)
Lost to follow-up, n (%)	0 (0%)	0 (0%)	7 (46.7%)
Full recovery without any new deficits, n (%)*	11 (61.1%)	1 (14.3%)	0 (0%)
Long-COVID, n (%)*	6 (33.3%)	6 (85.7%)	3 (37.5%)
Concentration problems, n (%)*	3 (17.6%)	5 (71.4%)	1 (12.5%)
Memory problems, n (%)*	3 (17.6%)	4 (57.1%)	2 (25%)
Chronic fatigue, n (%)*	0 (0%)	5 (71.4%)	2 (25%)
Speech/communication difficulties, n (%)*	0 (0%)	1 (14.3%)	0 (0%)
Change/loss of smell, n (%)*	0 (0%)	2 (28.6%)	0 (0%)
Change/loss of taste, n (%)*	1 (5.9%)	1 (14.3%)	0 (0%)
Muscle pain, n (%)*	2 (11.8%)	1 (14.3%)	0 (0%)
Difficulties in daily activites, n (%)*	3 (17.6%)	2 (28.6%)	1 (12.5%)
Deceased, n (%)	1 (5.6%)	0 (0%)	5 (62.5%)
In hospital during the ongoing study, n (%)*	0 (0%)	-	3 (37.5%)
*percentage value refers to the number of conducted follow-ups			

Supplementary Table 7: 13-months follow-up results.

Results per Neuro-COVID class of a 13-months post-COVID-19 follow-up performed using the modified COVID-19 Yorkshire Rehabilitation Screening (C19-YRS).
