# Supplementary material for: Severe Neuro-COVID is associated with peripheral immune signatures, autoimmunity and neurodegeneration: a prospective cross-sectional study
Source: Nat Commun. 2022 Nov 9;13:6777. doi: 10.1038/s41467-022-34068-0 (PMC9645766; doi:10.1038/s41467-022-34068-0)
Supplement: Supplementary file 3 — Description of Additional Supplementary Files [file 41467_2022_34068_MOESM3_ESM.pdf]

Description of Additional Supplementary Files

File Name: Supplementary Data 1
Description: Detailed routine CSF parameters for each COVID-19 patient.

File Name: Supplementary Data 2
Description: NMDS components.

File Name: Supplementary Data 3
Description: Log_2 scaled CSF/plasma ratio for each individual analyte and differences across study cohorts.

File Name: Supplementary Data 4
Description: Nomenclature of relevant proteins in the study.

File Name: Supplementary Data 5
Description: Marginalized individual protein values and contrasts between different groups.

File Name: Supplementary Data 6
Description: ROC-AUC analysis of individual proteins in different NeuroCOVID classes.

File Name: Supplementary Data 7
Description: Brain MRI and cranial CT results per Neuro-COVID class.

File Name: Supplementary Data 8
Description: Brain imaging questionnaire.

File Name: Supplementary Data 9
Description: Main demographics and clinical variables of the COVID-19 imaging group and the volumetric imaging control group.

File Name: Supplementary Data 10
Description: Mean GMV across voxels belonging to each structure label.

File Name: Supplementary Data 11
Description: Clinical trial protocol.
