# Supplementary material for: Severe Neuro-COVID is associated with peripheral immune signatures, autoimmunity and neurodegeneration: a prospective cross-sectional study
Source: Nat Commun. 2022 Nov 9;13:6777. doi: 10.1038/s41467-022-34068-0 (PMC9645766; doi:10.1038/s41467-022-34068-0)
Supplement: Supplementary file 14 — Supplementary Data 11 [file 41467_2022_34068_MOESM14_ESM.pdf]

**Systematic assessment of SARS-CoV-2 neurotropic capacity in modestly and critically ill patients, and patients who died from COVID-19**

| | |
|---|---|
| Research legislation: | Ordinance on human research with the exception of Clinical trials (HRO) [1]. |
| Type of Research Project: | Research project involving human subjects |
| Risk Categorisation: | Risk category B |
| Project Leader: | Prof. Dr. med. Dr. sc. nat. Gregor Hutter<br>Gregor.hutter@usb.ch |

## PROTOCOL SIGNATURE FORM

| | |
|---|---|
| Study Title | **Systematic assessment of SARS-CoV-2 neurotropic capacity in modestly and critically ill patients, and patients who died from COVID-19** |

The project leader (main center) and the investigator (at the local center/site) have approved the protocol version ***2.0 (dated 11.09.2020),*** and confirm hereby to conduct the project according to the protocol, the Swiss legal requirements [1,2], the current version of the World Medical Association Declaration of Helsinki [3] and the principles and procedures for integrity in scientific research involving human beings.

**Project leader (lead center/site)**

Site *Universitätsspital Basel, Spitalstrasse 21, CH-4031 Basel*

Name: Prof. Dr. med. Dr. sc. nat. Gregor Hutter

Date: 11.09.2020 _________________ Signature:

## TABLE OF CONTENTS

TABLE OF CONTENTS 2
GLOSSARY OF ABBREVATIONS 3
1 BACKGROUND AND PROJECT RATIONALE 4
2 PROJECT OBJECTIVES AND DESIGN 4
2.1 Hypothesis and primary objective (and if applicable also secondary objectives) 4
2.2 Primary and secondary endpoints 5
2.3 Project design 5
3 PROJECT POPULATION AND STUDY PROCEDURES 6
3.1 Project population, inclusion and exclusion criteria 6
3.2 Recruitment, screening and informed consent procedure 7
3.3 Study procedures 8
3.4 Withdrawal and discontinuation 9
4 STATISTICS AND METHODOLOGY 9
4.1. Statistical analysis plan 9
4.2. Handling of missing data 10
5 REGULATORY ASPECTS AND SAFETY 10
5.1 Local regulations / Declaration of Helsinki 10
5.2 Notification of safety and protective measures (HRO Art. 20) 10
5.3 Serious events (HRO Art. 21) 10
5.4 Procedure for investigations involving radiation sources 10
5.5 Amendments 10
5.6 End of project 10
5.7 Insurance 10
6 FURTHER ASPECTS 10
6.1 Overall ethical considerations 10
6.2 Risk-Benefit Assessment 11
6.3 *If applicable:* Rationale for the inclusion of vulnerable participants 11
7 QUALITY CONTROL AND DATA PROTECTION 12
7.1 Quality measures 12
7.2 Data recording and source data 12
7.3 Confidentiality and coding 13
7.4 Retention and destruction of study data and biological material 13
8 FUNDING / PUBLICATION / DECLARATION OF INTEREST 13
9 REFERENCES 13
If applicable: Appendix 1: Schedule of assessments **Error! Bookmark not defined.**

## GLOSSARY OF ABBREVATIONS

| | |
|---|---|
| *BASEC* | *Business Administration System for Ethical Committees* |
| *CRF* | *Case report form* |
| *FOPH* | *Federal Office of Public Health* |
| *HRA* | *Human Research Act* |
| *HRO* | *Ordinance on Human* |

# 1 BACKGROUND AND PROJECT RATIONALE

The world is currently facing a pandemic caused by a highly pathogenic coronavirus, SARS-CoV-2, that induces a disease called COVID-19 Originally, it started from the city of Wuhan in China, but meanwhile, the virus has rapidly spread around the world, infecting more than 4 million people and causing more than 300'000 deaths so far.

Little is known about the neurotropism of SARS-CoV-2. Several other coronaviruses in mouse models and patients however have the potential to cause severe encephalitis. There are reports of anosmia in a considerable number of patients suffering from COVID-19. This might also suggest CNS involvement. Further, the profound respiratory depression in critically ill patients might be related to central respiratory dysfunction, with an underlying affection of the brainstem. A detailed neurological workup of the severely ill patients with CSF analysis and MRI imaging has not been published so far, but reports from centers in northern Italy suggest that neurological symptoms in COVID-19 patients are more frequent than previously assumed. Very recently, a case of acute hemorrhagic encephalitis in a COVID-19 affected individual was reported in the journal *Radiology*. Microglia — the only brain-resident immune cells — play an essential role in antiviral defense of the brain. These cells are resident macrophages of the brain parenchyma. They express relevant endosomal and cytosolic pathogen pattern recognition receptors for detecting viral molecules and are capable of efficiently sensing viral pathogens *in vitro*. Reactive microgliosis, defined as an increase in microglial numbers and a change from ramified to amoeboid morphology, is observed in both patients and mice with viral encephalitis. Whether microglial reactivity is an appropriate response to viral invasion, or contributes negatively to disease, continues to provoke debates. One additional COVID-19 entry receptor, CD147 (basigin, BSG), is expressed mostly on endothelial cells, astrocytes, and to a lower level on microglia in the brain, whereas ACE2 has generally low expression levels (https://www.brainrnaseq.org/). Interestingly, CD147 is upregulated under certain pathological conditions such as stroke, leading to blood brain barrier disruption and leukocyte infiltration, and its inhibition led to better outcomes in a mouse model of ischemic brain damage. Peripheral immune responses such as cytokine storm or other yet to be determined factors might therefore equally lead to upregulation of this entry receptor and facilitate viral spread into the CNS. To investigate the tumor microenvironment in glioblastoma, a malignant brain tumor, we recently established a microglia-focused, brain-specific 50+ marker CODEX panel. Using this panel, we were able to quantify the brain microenvironment composition and -reaction to specific therapeutic conditions in unprecedented depth. We will repurpose this panel to assess the neuroinflammatory microenvironment in specific brain regions of deceased COVID-19 patients, and provide an integrative view on yet unknown factors that facilitate neurotropism by taking into consideration clinical, neuroimaging, high-throughput cerebrospinal and plasma targeted proteomics.

# 2 PROJECT OBJECTIVES AND DESIGN

## 2.1 Hypothesis and primary objective

We hypothesize that the microglia reaction or direct neurotropic effects of CNS COVID-19 might play a pivotal role in pathogenesis and deleterious brain stem dysfunction in critically ill patients.

The peripheral (cerebrospinal fluid and peripheral blood) cytokine response to SARS-CoV-2 might be critical for CNS affection and consecutive blood brain barrier disruption leading to brain-inherent neuroinflammatory reactions.

### 2.2 Primary and secondary endpoints

- Evaluation of neurological/mental state and specific scores
- Systematic CNS MRI imaging to document specific changes due to COVID-19 infection
- Plasma and CSF cytokine targeted proteomics analysis
- Peripheral blood leukocyte CyTOF analysis
- CODEX (high dimensional microscopy) analysis and viral load/in situ distribution analysis of defined regions on brain autopsy specimens

Primary endpoints of this project are the multidimensional integration of the analysis form the procedures described above and assessment of the correlation between the gained clinical data (MRI, mental/neurological state), the body fluid proteomic and mass-cytometric analysis (CSF and Plasma proteomics, peripheral blood mass cytometry) and the CODEX analysis of defined brain regions on autopsy specimens. This will enable us to identify biomarkers that potentially predict CNS involvement of COVID-19 disease and tailor respective treatments.

### 2.3 Project design

The project flowchart is depicted below. Patients with confirmed COVID-19 disease specifically hospitalized for this disease as well as out-patients will be included. After patient informed consent, we will perform a detailed clinical-neurological assessment followed by blood and lumbar puncture, and MRI analysis. In case of disease-related death (assuming a case fatality rate on the ICU of 50%), an autopsy by the institute of pathology will be performed, encompassing high dimensional analysis of the organs under investigation.

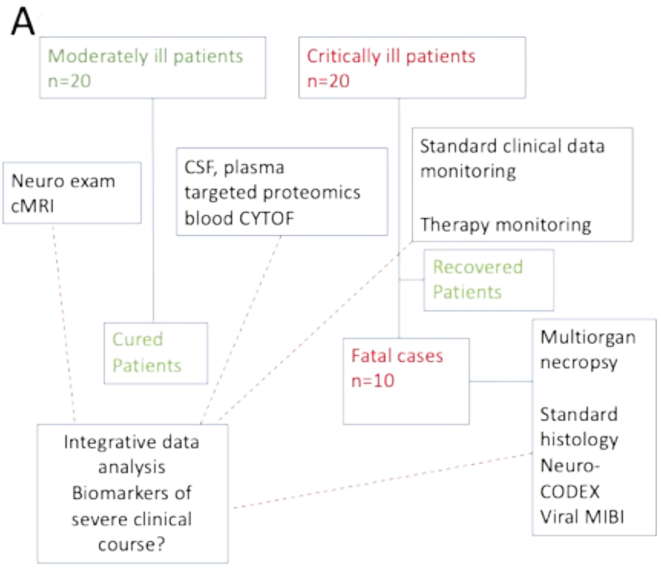


**CSF and plasma cytokine proteomics and correlate to mental/neurological state.** CSF will be acquired by lumbar puncture and sent for routine analysis including CSF chemistry, cytology and microbiology. Targeted CSF proteomics using OLINK Proximity Extension Assay (PEA) technology (https://www.olink.com/products/immune-response-panel/), to assess a potential CNS-contribution of the disease will be performed. A similar biomarker approach will be applied for plasma samples from the same time-point as the lumbar puncture. Already established CyTOF technology will be used to gain insight into the peripheral blood immune composition in both patient cohorts (CyTOF panel listed in table 3). Presence of viral RNA will be tested by our in-house qPCR in both plasma and CSF. We will include COVID-19 negative CSF and plasma samples from healthy reference patients into this analysis.

**CyTOF (mass cytometry analysis) of peripheral blood leukocytes** Already established CyTOF technology will be used to gain insight into the peripheral blood immune composition in both patient cohorts. Presence of viral RNA will be tested by our in-house qPCR in both plasma and CSF. We will include COVID-19 negative CSF and plasma samples from healthy reference patients into this analysis.

**Systematic CNS MRI imaging to document early/manifest encephalitic changes in COVID-19 patients.** Both patient populations will undergo 3 Tesla magnetic resonance imaging (MRI) in collaboration with our colleagues from neuroradiology (responsible: Dr. Johanna Lieb). MRI sequences will include 3D T1 +/- gadolinium, FLAIR, diffusion, SWI and T2 weighted sequences to document potential early signs of encephalopathy, and will be correlated to CSF, plasma and clinical findings. Appropriate precautions will be taken to ensure the safety in the MRI scanner of patients with respiratory, neurological, or other types of dysfunction, and all necessary anti-infectious precautions will be observed before, during, and after the scanning of patients with known COVID infection.

**High dimensional, microglia-centric CODEX fluorescent microscopy of defined brain regions on autopsy specimens from medulla oblongata, cortex, cerebellum, and olfactory bulb.** We recently implemented a brain-specific CODEX panel for the assessment of the immune microenvironment, with inclusion of a wide array of myeloid/microglia markers, T-cell markers, and a few neuronal markers. All the antibodies have been validated and tested in formalin-fixed tissues of different zones of glioblastoma tumors including invading tumor periphery. The panel will be modified to include the viral entry receptors, CD147, ACE2 and TMPRSS2. Presence of SARS-CoV-2 virus particles will be assessed by qPCR, and MIBI technology will be used to visualize tropism of virus to specific cellular brain compartments. The downstream processing pipeline (segmentation, clean-up gating, clustering, neighborhood analysis, visualization and high-performance computing) have been established at our institution in the last year in the framework of a project funded by The Brain Tumour Charity, UK (grant number GN-000562).

Microglia reaction patterns, orchestrated with other CNS immune cells and in interaction with neuronal and glial counterparts, might play a crucial role in determining the severity of COVID-19, ultimately leading to central respiratory failure. We will be able to assess a multitude of phenotypic characteristics of the tissue reaction and neuronal/glial/immune cell crosstalk at once. Moreover, by having access to a wealth of clinical data including MRI images and CSF proteomics, we expect to find indicators/biomarkers for a worse clinical course resulting from microglial or other CNS-immune cell-mediated overactivity and/or neuronal death, which could be counteracted with CNS-specific treatments in the future.

# 3 PROJECT POPULATION AND STUDY PROCEDURES

## 3.1 Project population, inclusion and exclusion criteria

We will *prospectively* include 20 non-critically ill COVID-19 patients from the inpatient ward or from the ambulatory test centre, and 20 critically ill COVID-19 patients needing mechanical ventilation at the ICU. Neurological assessment will be performed by a board-certified neurologist using NIHSS (National institute of Health Stroke Scale) scoring and other standard neurological tests. Neurological symptoms will be carefully documented. Assuming a case fatality rate of 50% in the critically ill group, we anticipate to include 10 patients for *post mortem* analysis of brain regions, some of which might be neurologically affected.

The following inclusion criteria are going to be applied: COVID-19 positive tested.

The following exclusion criteria are going to be applied: COVID-19 negative tested, patients under 18 years old, pregnant women.

A pregnancy test is performed and documented for women of childbearing age

Concerning the out-patients they will only be included if they have the option to come to the hospital by car, bike or if they live close enough to walk. In case public transport is their only option they can not be included.

To avoid conflicts and to identify parallel participation of individual USB patients in several studies, the DKF (Departement Klinische Forschung) has created a web application.

### 3.2 Recruitment, screening and informed consent procedure

Recruitment takes places at University Hospital of Basel. Critically ill patients are going to be recruited at the Intensive Care Unit (ICU), modestly ill patients are going to be recruited from "normal" hospital ward through the project leader.

In case of hospitalized patients that can not be informed, we are going to inform their relatives about the study procedures.

Recruitment procedure is supported from clinical departments of the ICU (Prof. H. Pargger) (critically ill patients) and Internal Medicine (Prof. S. Bassetti) (modestly ill patients).

Patients who got tested COVID-19 positive in the ambulatory test centre of the University Hospital of Basel will be handed an information sheet about this project including our contact information. Should they be interested in participating, they can send an email including their name, surname, phone number and date of birth to an email account specifically created for this project (neurocovid@usb.ch). Afterwards they will get a phone call, where they will be informed in detail about the procedures that take place and their risks and benefits. If desired we are also able to organize a skype or zoom call.
If they agree, they will receive the patient information and agreement via email or by post and an appointment date will be set for the study procedures. The study agreement has to be printed and signed. The out-patients will have to bring it along to the appointment date for the study procedures. In case they are not in charge of a printer or they forget to bring the signed agreement along, we prepare printed versions of the information and agreement sheet. Then it can be read and signed directly at the University Hospital of Basel during the appointment. For this case we include an extra hour before study procedures start. During this time the out-patients can go through the information again and ask further questions.
Because the out-patients incur a hospital stay only for their participation in this project they will get paid with an amount of 200 CHF.

Concerning the commute to the hospital, we will give precise advice as to how to reach the hospital. We will suggest to take the car, walk or take the bike. Also they will be informed to wear a mask at all times as soon as they leave the house. Should public transport be their only option, they will be excluded from the study. These advices apply for the way home as well.

For informed consent process, the study is going to be explained to the potential participant verbally, providing all pertinent information (purpose, procedures, risks, benefits etc.) with ample opportunity to ask questions.

Following the verbal explanation, the potential participant is going to be provided with a study information sheet. To consider whether or not to participate in this research sufficient time is going to be afforded (adapted on how long it reasonably takes to evaluate the procedures, risks, benefits etc.).

After the potential participant had time to read the study information sheet, the Investigator is going to answer any additional questions. For participation in the research the participates agreement is going to be recorded on the study information sheet. The potential participants will have 24h time to decide their participation.

In case of potential participants who are unable to listen, read and write, the study is going to be explained to their relatives. We will inform the relatives by telephone about this project and make an appointment (at University Hospital of Basel) for informed consent process.

In case of death, the relatives of the participant are going to be asked for consent to perform autopsy at the institute of pathology, University Hospital of Basel. The relatives are going to be are going to be contacted by telephone to make an appointment (at University Hospital of Basel) for informed consent process regarding the autopsy regulations at the institute of pathology. Informed consent process for autopsy takes place in accordance with the standard procedure of the University Hospital of Basel.

### 3.3 Study procedures

Overall project duration is 2.5 years, while recruitment period of participants is scheduled for 1 year. Project start is planned for July 2020 after approval by the ethics committee, end of the study is planned for 31 December 2022. Depending on the situation it is planned to involve further sites in Switzerland. Project duration for each patient takes 1 hour for the MRI, 30 minutes for the lumbar puncture and 10 minutes for blood withdrawal. For the hospitalized patients each of the described procedures takes place during hospitalization period. Thus, the participants do not have extra visits at the hospital. Concerning the out-patients we are going to set an appointment for the procedures. The appointment should take place as soon as possible after they received their positive test results, thus during their isolation period. The described procedures are going to take place in a prepared isolation room at the University Hospital of Basel at Klinikum 1 where usually consultation hours take place. The isolation room will be labeled as such and protection clothing will be put in a locker in front of the room. The MRI procedure will be taking place in the same manner as for the hospitalized patients. For precautionary purpose, there will only be one patient at a time and after each appointment the rooms will be cleaned and disinfected according to the usual room cleaning procedures after a hospital stay of COVID-19 infected patients. The appointment will take place during a flexible time band to minimalize contact with other people at the hospital and outside.

Material from blood withdrawal and lumbar puncture is going to be frozen at -80° C and stored at the Department of Biomedicine, 4$^{th}$ floor, lab 414, University Hospital Basel, until analysis. Clinical data (neurological examination, neurological scores, laboratory parameters, MRI imaging data) are saved in the clinical information system (ISMED,University Hospital of Basel program for patient's data, and the ICU's proprietary data documentation system) and for further analysis exported in an encoded form to a specific location on the telemedicine software ipath (www.ipath-network.com/usbnch), which is already in use for the brain tumor data base of the neurosurgical clinic, University Hospital Basel (please refer to REQ-2019-00553). Only designated persons specifically employed for this project will have access to the clinical data saved on this platform.

The autopsy of specific brain regions takes place at the institute of pathology at University Hospital Basel. The tissue of the specific brain regions will be fixed, paraffin embedded and stored at the Department of Biomedicine 4$^{th}$ floor, lab 414, University Hospital Basel, until analysis. For analysis the tissue is subjected to the CODEX workflow by staining with a 50+ Marker panel. This is followed by a complex analysis pipeline which generates single-cell information of specific cell types derived from the multicycle image acquisition.

**Exact description of the body parts**

For this project, specific brain regions of COVID-19 infected and deceased persons, included in this study, are investigated on autopsy specimens from medulla oblongata, cortex, cerebellum, and olfactory bulb. We will include only tissue samples, which have been submitted to the Institute of Pathology, University Hospital Basel.

**Ban on commercialisation (Art. 9 HFG)**

The bodies or parts of bodies shall not be sold or taken into consideration for any other monetary benefit.

### 3.4 Withdrawal and discontinuation

Patients can withdraw their consent at any time without giving reasons. There will be no negative consequences. After withdrawal, the collected data and samples remain encrypted. We will use data and the samples until this time point for analysis otherwise the project will lose his value.

## 4 STATISTICS AND METHODOLOGY

### 4.1. Statistical analysis plan

This study encompasses a multidimensional exploratory data set of both clinical and biological data. In this Project we are going to collect data sets of 40 participants.

*MRI data:* Imaging data will be analysed by board certified neuroradiologists, and specific encephalitic changes that might be COVID-19 related, will be reported and conjoined with potential neurological symptoms depending on the location of the potential encephalitic lesions. Most importantly, patients that are neurologically affected will be compared to non-affected individuals in terms of CNS involvement.

*Proteomic analysis:* The proteomic biomarkers from an array of cytokines and other inflammatory mediators (CSF and plasma, provided by the company Olink.com) will be compared to control reference sample using descriptive statistics and multivariate comparisons such as clustering algorithms, heatmaps, and correlative analysis. Furthermore, potential treatment effects from antiviral drugs or immune modulators might be taken into considerations.

*Cytof analysis*: Mass cytometry will be performed form peripheral blood mononuclear cells and analyzed with specific platoforms such as immune-Atlas and FlowJo software. High dimensional nearest neighbourhood analysis will be performed (tSNE plots). Cell population frequency and marker expression profile will be potentially correlated to the presence/absence of neurological symptoms and the respective cytokine profile.

*CODEX analysis*: Brain region from deceased patients will be fixed, paraffin embedded and subjected to the CODEX workflow by staining with a 50+ Marker panel. This is followed by a complex analysis pipeline which generates single-cell information of specific cell types derived from the multicycle image acquisition. Images will be segmented, cleaned-up, and cells will be clustered in supervised and unsupervised manner. Global and single marker expression per cell type will be assessed. Cellular neighbourhoods combined with viral distribution will be analyzed. This pipeline is currently implemented at the Department of Biomedicine and pathology and subject to ongoing modifications.

### 4.2. Handling of missing data

We assume to have complete data from blood, CSF and MRI analysis from all the participating patients. If for some reason the dataset is not complete, this will be reported in the data analysis.

## 5 Regulatory Aspects and Safety

### 5.1 Local regulations / Declaration of Helsinki

This research project will be conducted in accordance with the protocol, the Declaration of Helsinki [3], the principles of Good Clinical Practice, the Human Research Act (HRA) and the Human Research Ordinance (HRO) [1] as well as other locally relevant regulations. The Project Leader acknowledges his responsibilities as both the Project Leader and the Sponsor.

### 5.2 Notification of safety and protective measures (HRO Art. 20)

The project leader is promptly notified (within 24 hours) if immediate safety and protective measures have to be taken during the conduct of the research project. The Ethics Committee will be notified via BASEC of these measures and of the circumstances necessitating them within 7 days.

### 5.3 Serious events (HRO Art. 21)

If a serious event occurs, the research project will be interrupted and the Ethics Committee notified on the circumstances via BASEC within 7 days according to HRO Art. 21[1].

### 5.4 Procedure for investigations involving radiation sources

### 5.5 Amendments

Substantial changes to the project set-up, the protocol and relevant project documents will be submitted to the Ethics Committee for approval according to HRO Art. 18 before implementation. Exceptions are measures that have to be taken immediately in order to protect the participants.

### 5.6 End of project

Upon project completion or discontinuation, the Ethics Committee is notified within 90 days.

### 5.7 Insurance

In the event of project-related damage or injuries, the Sponsor will be liable, except for damages that are only slight and temporary; and for which the extent of the damage is no greater than would be expected in the current state of scientific knowledge (Art. 12 HRO).
Because this project is a category B research project, an additional insurance package is assured.

## 6 FURTHER ASPECTS

### 6.1 Overall ethical considerations

---

[1] A serious event is defined as any adverse event where it cannot be excluded, that the event is attributable to the sampling of biological material or the collection of health-related personal data, and which:
a. requires inpatient treatment not envisaged in the protocol or extends a current hospital stay;
b. results in permanent or significant incapacity or disability; or
c. is life-threatening or results in death.

Very little is known about COVID-19 pathobiology, its effects on human tissue, its tropism in the human body and its interactions with the immune system. Besides, almost no data exist about the tropism of SARS-CoV-2 within the human body. Although the respiratory tract is thought to be the main virus reservoir, it remains unknown which additional cells of the body are being infected and to what consequences this leads. Moreover, the virus' interaction with the immune system is still poorly characterized, although being particularly important.

Therefore, it is of major importance to identify the mechanisms of SARS-CoV-2 which are leading to infection, severe illness and death. Based on these findings we could identify markers predictive for a severe course. Furthermore, new therapeutic approaches could be generated based on the pathophysiologic mechanisms of this virus, with the result of beneficial treatment response in patients infected with SARA-CoV-2.

In case of incidental findings (MRI images, lumbar puncture, blood withdrawal, neurological state/scores) the participants are informed.

### 6.2 Risk-Benefit Assessment

Risks of the procedures in this project:

- MRI: allergic reaction to contrast agents
- Lumbar puncture: Infection, post-lumbar puncture headache
- Blood withdraw: Infection.

Each of the mentioned risks takes place in less than 1%. To reduce the risk of infection the procedures of lumbar puncture and blood withdraw are kept under sterile conditions (disinfection, sterile gloves, protective masks etc.). During MRI procedure the participant is observed by specialized employees.

Results of the project could benefit future patients infected with SARS-CoV-2. We could better understand the mechanisms that take place during infection with SARS-CoV-2, identify predictive markers for severe course and building on these findings new treatment approaches could be generated.

### 6.3 Rationale for the inclusion of vulnerable participants

We will include participants in this project, who are unable to listen, read and write. In this case, the study is going to be explained to their relatives. We will inform the relatives by telephone about this project and make an appointment (at University Hospital of Basel) for informed consent process.

For informed consent process, the study is going to be explained to the relatives of potential participant verbally, providing all pertinent information (purpose, procedures, risks, benefits etc.) with ample opportunity to ask questions.

Following the verbal explanation, the relatives of the potential participant are going to be provided with a study information sheet. To consider whether or not to participate in this research sufficient time is going to be afforded (adapted on how long it reasonably takes to evaluate the procedures, risks, benefits etc.).

After the relatives of the potential participant had time to read the study information sheet, the Investigator is going to answer any additional questions. For participation in the research the relatives agreement is going to be recorded on the study information sheet.

After obtaining the ability to judge, we will let the participants subsequently agree to participate in the study.

# 7 QUALITY CONTROL AND DATA PROTECTION

## 7.1 Quality measures

Our valuable tissue will be analyzed extensively by various and complementary methods to improve our knowledge of this disease, in particular its pathogenic effect on human tissue, its tropism in the human body and its interactions with the immune system and the microcirculation. Moreover, we can bring in all our expertise in autopsy and histological sample evaluation that we have collected over the past years and generate data of exceptional value for the scientific community.

For an in-depth insight into the immune reaction, we will collaborate with the Nolan laboratory at Stanford University (USA). The Nolan laboratory is a worldwide leader in performing high-parameter, single-cell analyses to simultaneously enumerate and characterize the activation state of a vast number of immune cells. They have developed important technologies including CyTOF, MIBI and CODEX, and numerous computational algorithms high-parameter data analysis from across a variety of assays, critical for deep analysis of immune phenotypes and tissue pathology at scale. We are currently setting up the CODEX technology at the University of Basel together with other research groups and the necessary instruments to perform this technique are available. However, given the urgent need for quick results in the COVOID-19 pandemic, we will collaborate with our colleagues at Stanford University, where these technologies are already well-established and all the expertise is available. Moreover, the Hutter laboratory already has performed a CODEX project in collaboration with the Nolan laboratory and has established the downstream analysis pipeline. Every other technique for the goals of the present grant is established at our institute with the respective know-how.

Finally, we will work closely with the clinical departments of the Intensive Care Unit (Prof. H. Pargger), Internal Medicine (Prof. S. Bassetti), Pneumology, Infectious disease unit and Neuroradiology/Neurology of the University Hospital Basel. This collaboration will allow us to correlate our *in situ* r esults from the tissue with the clinical data and the treatments given to the patients. This is particularly important with regard to discovering proper clinical management strategies to improve the prognosis of COVID-19 patients.

For quality assurance the Ethics Committee may visit the research sites. Direct access to the source data and all project related files and documents must be granted on such occasions.

## 7.2 Data recording and source data

The data of interest in this project is gained from MRI images, CSF, blood plasma and documentation of neurological/mental state/scores. These procedures take only place in case of agreement to this research project. Not all the critically ill COVID-19 patients at ICU have to undergo MRI imaging, evaluation of neurological state and lumbar puncture. According to clinical routine, blood withdrawal is already done.

The documented clinical findings (neurological/mental state/sore, MRI images and laboratory parameters) are saved in the University Hospital of Basel software Ismed. Additionally, data relevant for the project (especially basic lab parameters, clinical course, neurological findings including EEG, scores, MRI imaging) is saved in an encoded form in an online repository also used for the brain tumor database of the University Hospital Basel (ipath-network.com/usbnch). Only designated coworkers for this project will have access to the data.
The acquired fluids of lumbar puncture, blood withdrawal and autopsy (in case of death) are going to be frozen at -80° C and stored at Department of Biomedicine, 4$^{th}$ floor, Lab 414, University Hospital of Basel.

All ambulatory patients responding to our information sheet will be listed in an excel file which is saved in an encoded form in an online repository also used for the brain tumor database of the University Hospital Basel (ipath-network.com/usbnch). Only designated coworkers for this project will have access to the data.

### 7.3 Confidentiality and coding

**Project data** will be handled with utemost discretion and is only accessible to authorized personnel who require the data to fulfil their duties within the scope of the research project. On the CRFs and other project specific documents, participants are only identified by a unique participant number.
For this project coded non-genetic data is used. Data protection is obtained by using a secure and encrypted system for the storage of the data. The assignment of a standardized ID takes place at patient inclusion date upon signature of the informed consent form. Only selected, active coworkers involved in this project have password-protected access to the data which is stored at an online repository (ipath-network.com/usbnch) and servers of the Department of Biomedicine. Employees which leave the University Hospital of Basel are going to be excluded from the encrypted system. The data is not irreversibly encrypted. In case of significant and relevant findings we are able to draw on the true identity of the participant. The list of patients with the respective code is securely stored in the office of study nurse Heike Neddersen, Neurosurgery Department. The gained data and findings are only transferred in an encrypted form. Publication of the data does not enable drawing on the true identity of participants included in this project.

**Biological material** in this project is not identified by participant name but by a unique participant number. Biological material is appropriately stored in a restricted area only accessible to authorized personnel.
For this project coded non-genetic data is used. Data protection is obtained by using a secure and encrypted system for the storage of the data. The award of a standardized ID takes place before the procedures of the project take place (lumbar puncture etc.). Only selected, active employees of the University Hospital of Basel have password-protected access to the data. Employees which leave the University Hospital of Basel are going to be excluded from the encrypted system. The data is not irreversibly encrypted. In case of significant and relevant findings we are able to draw on the true identity of the participant. The gained data and findings are only transferred in an encrypted form. Publication of the data does not enable drawing on the true identity of participants included in this project.

### 7.4 Retention and destruction of study data and biological material

The data is stored at the biobank of the Neurosurgical Department, Department of Biomedicine, Lab 414, 4$^{th}$ floor, for 10 years. The storage of the gained data takes place with the standards and principles of the Brain Tumor Biobank regulations.

## 8 FUNDING / PUBLICATION / DECLARATION OF INTEREST

This project is funded by the Botnar Research Centre for Child Health. After this project, the aim is to publish our findings. We have no competing financial interests in the frame of this project.

## 9 REFERENCES

1. Ordinance on Human Research with the Exception of Clinical trials (HRO) https://www.admin.ch/opc/en/classified-compilation/20121177/index.html
2. Human Research Act (HRA) http://www.admin.ch/opc/en/classified-compilation/20121176/201401010000/810.305.pdf

3. Declaration of Helsinki (https://www.wma.net/policies-post/wma-declaration-of-helsinki-ethical-principles-for-medical-research-involving-human-subjects )
4. STROBE statement (http://www.jclinepi.com/article/S0895-4356(07)00436-2/pdf)
