# Supplementary material for: Severe Neuro-COVID is associated with peripheral immune signatures, autoimmunity and neurodegeneration: a prospective cross-sectional study
Source: Nat Commun. 2022 Nov 9;13:6777. doi: 10.1038/s41467-022-34068-0 (PMC9645766; doi:10.1038/s41467-022-34068-0)
Supplement: Supplementary file 15 — Reporting Summary [file 41467_2022_34068_MOESM15_ESM.pdf]

nature portfolio

Corresponding author(s): Gregor Hutter

Last updated by author(s): 28.09.2022

Reporting Summary

Nature Portfolio wishes to improve the reproducibility of the work that we publish. This form provides structure for consistency and transparency in reporting. For further information on Nature Portfolio policies, see our Editorial Policies and the Editorial Policy Checklist.

Statistics

For all statistical analyses, confirm that the following items are present in the figure legend, table legend, main text, or Methods section.

n/a	Confirmed	
☐	☒	The exact sample size (*n*) for each experimental group/condition, given as a discrete number and unit of measurement
☐	☒	A statement on whether measurements were taken from distinct samples or whether the same sample was measured repeatedly
☐	☒	The statistical test(s) used AND whether they are one- or two-sided<br>*Only common tests should be described solely by name; describe more complex techniques in the Methods section.*
☐	☒	A description of all covariates tested
☐	☒	A description of any assumptions or corrections, such as tests of normality and adjustment for multiple comparisons
☐	☒	A full description of the statistical parameters including central tendency (e.g. means) or other basic estimates (e.g. regression coefficient) AND variation (e.g. standard deviation) or associated estimates of uncertainty (e.g. confidence intervals)
☐	☒	For null hypothesis testing, the test statistic (e.g. *F*, *t*, *r*) with confidence intervals, effect sizes, degrees of freedom and *P* value noted<br>*Give P values as exact values whenever suitable.*
☒	☐	For Bayesian analysis, information on the choice of priors and Markov chain Monte Carlo settings
☐	☒	For hierarchical and complex designs, identification of the appropriate level for tests and full reporting of outcomes
☒	☐	Estimates of effect sizes (e.g. Cohen's *d*, Pearson's *r*), indicating how they were calculated

Our web collection on statistics for biologists contains articles on many of the points above.

Software and code

Policy information about availability of computer code

Data collection

No software was used for data collection.

Data analysis

Volumetric brain imaging analysis:
- SPM software (freely available) is a suite of MATLAB (MathWorks) functions and subroutines with some externally compiled C routines (Wellcome Trust Centre for Neuroimaging, London, UK; http://www.fil.ion. ucl.ac.uk/spm).
- Neuromorphometric atlas: SPM12 introduces a new atlas “labels_Neuromorphometrics.” (see https://github.com/neurodebian/spm12/blob/master/spm_templates.man) (http://Neuromorphometrics.com/)
About this atlas: Maximum probability tissue labels derived from the “MICCAI 2012 Grand Challenge and Workshop on Multi-Atlas Labeling” are available in files tpm/labels Neuromorphometrics.{nii,xml}. These data were released under the Creative Commons Attribution-NonCommercial (CC BY-NC) with no end date. The MRI scans as originating from the OASIS project and the labeled data as “provided by Neuromorphometrics, Inc. under academic subscription”.

B cell receptor sequencing data analysis:
CDR3 nucleotide length of each clone was calculated and CDR3 length distribution was plotted with R Studio Version 4.1.2. ggplot 2.

Statistical data analysis of any CSF and plasma data:
All used data analysis softwares/tools/algorithms/packages are accessible under this link: https://github.com/WandrilleD/severe-neuro-COVID-cross-sectional-study-etteretal2022/blob/master/etter_env.yml
In short the following were used: python 3.8.8, matplotlib 3.5.2, pandas 1.4.2, scikit-learn 0.24.1, seaborn 0.11.1, statsmodels 0.13.2, numpy 1.21.6, scipy 1.8.0., R version 4.1.2 (R Core Team 2021) and the following R packages: BiocStyle v. 2.22.0 (Olés 2021), caret v. 6.0.92 (Kuhn 2022), circlize v. 0.4.14 (Gu et al. 2014), coefplot v. 1.2.8 (Lander 2022), ComplexHeatmap v. 2.10.0 (Gu, Eils, and Schlesner 2016), cvms v.

1.3.4 (Olsen and Zachariae 2022), factoextra v. 1.0.7 (Kassambara and Mundt 2020), futile.logger v. 1.4.3 (Rowe 2016), ggpubr v. 0.4.0 (Kassambara 2020), glmnet v. 4.1.4 (Friedman, Hastie, and Tibshirani 2010; Simon et al. 2011), glmnetcr v. 1.0.6 (Archer and Williams 2012), grateful v. 0.1.11 (Rodriguez-Sanchez, Jackson, and Hutchins 2022), InformationValue v. 1.2.3 (Prabhakaran 2016), kableExtra v. 1.3.4 (Zhu 2021), knitr v. 1.40 (Xie 2014, 2015, 2022), MASS v. 7.3.57 (Venables and Ripley 2002), plotmo v. 3.6.2 (Milborrow 2022), plotROC v. 2.3.0 (Sachs 2017), pROC v. 1.18.0 (Robin et al. 2011), rmarkdown v. 2.16 (Xie, Allaire, and Grolemund 2018; Xie, Dervieux, and Riederer 2020; Allaire et al. 2022), tidyverse v. 1.3.1 (Wickham et al. 2019), umap v. 0.2.8.0 (Konopka 2022), UpSetR v. 1.4.0 (Gehlenborg 2019), VennDiagram v. 1.7.3 (Chen 2022).

For manuscripts utilizing custom algorithms or software that are central to the research but not yet described in published literature, software must be made available to editors and reviewers. We strongly encourage code deposition in a community repository (e.g. GitHub). See the Nature Portfolio guidelines for submitting code & software for further information.

Data

Policy information about availability of data

All manuscripts must include a data availability statement. This statement should provide the following information, where applicable:

- Accession codes, unique identifiers, or web links for publicly available datasets
- A description of any restrictions on data availability
- For clinical datasets or third party data, please ensure that the statement adheres to our policy

All data underlying the study are available within the submitted manuscript. No restrictions on data availability such as a materials transfer agreement are foreseen for this study. The trial protocol are available upon request from the corresponding author. The final trial protocol is provided as a supplementary file. Source data are provided with this paper as an .xlsx file with specified different sheets corresponding to the Main Figures, Supplementary Figures and Supplementary Tables provided with this manuscript.

Human research participants

Policy information about studies involving human research participants and Sex and Gender in Research.

Reporting on sex and gender	We included both male and female patients in this cross-sectional prospective trial. Accordingly, the control cohorts were age- and sex-matched. All proteomic analysis provided in this paper were marginalized according to age and sex, and reported in marginalization report in the github link: https://github.com/WandrilleD/severe-neuro-COVID-cross-sectional-study-etteretal2022. Gender issues were not considered in the data analysis.
Population characteristics	Inclusion criteria were a minimum age of 18 years and a real-time quantitative PCR (qRT-PCR)-positive SARS-CoV-2 infection. The only exclusion criteria was pregnancy. If patients were not able to provide informed consent, informed consent was provided by their relatives.
Recruitment	Patients were recruited during a period from August 2020 to April 2021 at two sites, the University Hospitals Basel and Zurich. Patients were recruited at the COVID-19 test center, the hospital ward or at the intensive care unit. Out of 40 patients, 35 donated paired blood and CSF samples, whereas 5 participants donated only blood samples. For their additional hospital visit, patients recruited at the test center were paid 200 swiss francs. Patients were properly informed about the rationale behind this study, the procedures with all the possible risks and the possible benefit for the general population as a result of this study. We provided each potential participant an information sheet explaining in written form the rationale of the study, planed procedures, risks of the mentioned procedures and potential benefit for the general population. If patients were not sure about whether to participate or not, they had time to think about their potential participation and the opportunity to contact us by email or phone. Patients who were tested in the COVID-19 test center at the University hospital Basel received a flyer shortly presenting the study and the same information file as the hospitalized patients. If they were interested in participating they contacted us by email or phone. Then the outpatients had the opportunity to ask questions and get more informations and explanations about study details before conforming their participation. After they confirmed their participation, we scheduled a date for their additional hospital visit to perform a neurological exam, a brain MRI, a lumbar puncture and the blood withdrawal. Lumbar puncture and blood withdrawal took place under sterile conditions in an isolated room at the outpatients clinic.
Ethics oversight	The study was approved by the Ethics Committee of Northwestern and Central Switzerland (clinicaltrials.gov NCT04472013, IRB approval EKNZ 2020-01503).

Note that full information on the approval of the study protocol must also be provided in the manuscript.

Field-specific reporting

Please select the one below that is the best fit for your research. If you are not sure, read the appropriate sections before making your selection.

☒ Life sciences ☐ Behavioural & social sciences ☐ Ecological, evolutionary & environmental sciences

For a reference copy of the document with all sections, see nature.com/documents/nr-reporting-summary-flat.pdf

Life sciences study design

All studies must disclose on these points even when the disclosure is negative.

Sample size	We did not use statistical methods to pre-determine sample sizes, but our sample sizes are similar or higher to those reported in previous publications. We aimed at prospectively recruiting 40 high-quality, CSF and plasma samples, and MRI datasets in conjunction with biobanked, clinically annotated control samples for this exploratory analysis.
Data exclusions	No data was excluded from the statistical analysis.
Replication	Using our co-submitted downstream data analysis code package, the reported results can be replicated. Proteomic analysis of additional CSF and plasma samples in another set of study patients with similar clinical conditions would enable replication of the results. In the meantime, vaccination of patients, and occurence of other Sars-CoV-2 virus strains might be factors that should be taken into consideration.
Randomization	Randomization or allocation of patients was not relevant for this study. We did not attempt to try new therapeutical interventions (e.g. medications) or diagnostics (e.g. new imaging modalities). Also, we did not intend to compare any existing therapeutics or diagnostics, which would have made randomization eligible. We attempted to identify severity dependent immune alterations and pathomechanisms in COVID-19 patients which suffered from different severity stages and to describe our findings in a descriptive form.
Blinding	As this is a cross-sectional study, blinding for patient allocation to different severity classes was not applicable. Patient allocation to severity classes made it necessary to assess the clinical symptoms of each patient (obtaining the medical history and performing neurological examination) and therefore blinding was not applicable or possible. Also, we did neither intend to test new therapeutic or diagnostic modalities nor to compare different treatments or diagnostic procedures. We aimed at describing the results of our study without a linkage to the participants' COVID-19 treatment medications and/or diagnostic modalities.

Reporting for specific materials, systems and methods

We require information from authors about some types of materials, experimental systems and methods used in many studies. Here, indicate whether each material, system or method listed is relevant to your study. If you are not sure if a list item applies to your research, read the appropriate section before selecting a response.

Materials & experimental systems

n/a	Involved in the study	
☐	☒	Antibodies
☒	☐	Eukaryotic cell lines
☒	☐	Palaeontology and archaeology
☒	☐	Animals and other organisms
☐	☒	Clinical data
☒	☐	Dual use research of concern

Methods

n/a	Involved in the study	
☒	☐	ChIP-seq
☒	☐	Flow cytometry
☐	☒	MRI-based neuroimaging

Antibodies

Antibodies used	- Humanized MOG- (h818C5) monoclonal: Not commercially available, kindly provided by Klaus Dornmair (unpublished).<br>- NF155-specific (A12/18.1) monoclonal: Not commercially available, kindly provided by Christopher Linington (Reference: Mathey, E.K. et al. Neurofascin as a novel target for autoantibody-mediated axonal injury. The Journal of Experimental Medicine 204, 2363-2372 (2007).<br>- ED38: Not commercially available, kindly provided by Hedda Wardemann. Reference: Wardemann, H. et al. Predominant autoantibody production by early human B cell precursors. Science 301, 1374–1377 (2003).
Validation	Validation of each primary antibody was performed at the respective laboratory that provided the antibody, see respective reference.

Clinical data

Policy information about clinical studies

All manuscripts should comply with the ICMJE guidelines for publication of clinical research and a completed CONSORT checklist must be included with all submissions.

Clinical trial registration	NCT04472013
Study protocol	The study protocol can be assessed in the supplementary material.
Data collection	Patient recruitment took place at two sites, the University Hospital Zurich (intensive care unit/stroke unit) and the University Hospital of Basel (normal hospital ward, intensive care unit, outpatients COVID-19 test center). Patients were recruited during the time period

from August 2020 and April 2021.
Lumbar puncture (LP) and blood withdrawal were performed concomitantly, on an average latency period of 4 days after the first positive SARS-CoV-2 qRT-PCR test result. For hospitalized patients on the intensive care unit, stroke unit or hospital ward the LP was performed in their respective isolated patient room. LP was performed under sterile conditions using a 20 gauge needle under local anesthesia on lumbar midline levels L4/5. Patients were monitored for positional headache or signs of CSF leakage for 24 h after puncture. Blood withdrawal mostly took place on the same day, but with a maximum latency period of 4 days. Blood withdrawal was performed under clinical standard conditions in the patient room for patients who were already hospitalized. The MRI/CT scan was performed during their hospital stay at fastest possible date. In case of intensive care unit patients the latency period of LP/blood withdrawal and brain scans was up to 10 days, due to logistical and staff reasons (these patients had to be monitored during their way to the MRI/CT, during the whole brain scan period and during their way back to the intensive care unit).
For patients recruited from the test center in Basel, a specific date was arranged for their hospital visit and data collection. The LP and blood withdrawal were performed on the same day for outpatients who came to the hospital for study reasons only. These patients were accommodated in an isolated COVID-19 room on the internal medicine outpatients clinic at University hospital Basel. There the previously described procedures took place (LP under sterile conditions).

Outcomes

This is an exploratory/cross-sectional study. Outcomes are to identify associations of COVID-19 and Neuro-COVID severity with a targeted set of CSF and plasma biomarkers, and structural neuroimaging, as well as possible impact of these biomarkers on long-COVID development.
Outcomes were assessed through allocation of patients to different severity stages of their clinical neurological symptoms and the consequently performed procedures, including LP, blood withdrawal, structural and volumetric brain imaging, and a 13-months patient reported follow-up. The results of the targeted CSF and plasma protein set were described for each severity stage of Neuro-COVID to identify different patterns of CSF and plasma proteins possibly characterizing different severity stages. Targeted CSF and plasma protein sets were also used for a AUC-ROC analysis to assess if there exists a set of potential CSF/plasma biomarkers to predict class III development (severe Neuro-COVID) and to predict long-COVID. AUC-ROC analysis of long-COVID biomarkers was performed using the information of the 13-months follow-up to investigate which patients suffer from long-COVID.
Structural MRI/CT scans were performed, analyzed and described in detail to investigate visible brain changes during acute inflammation and their association with different severity stages in Neuro-COVID. The volumetric MRI analysis was performed with a subset of Neuro-COVID patients (since a specific MRI sequence is necessary) and compared to a healthy control group to investigate whether Neuro-COVID patients of any severity stage display volumetric regional brain alterations. Volumetric brain alterations where then correlated with routinely assessed inflammatory CSF parameters to investigate whether there is an association between routinely measurable inflammatory CSF parameters and volumetric brain changes.

Magnetic resonance imaging

Experimental design

Design type

Only anatomical images were collected. N/A

Design specifications

N/A

Behavioral performance measures

N/A

Acquisition

Imaging type(s)

Structural brain imaging

Field strength

1.5 Tesla, 3 Tesla

Sequence & imaging parameters

3D T1-weighted (T1w) +/- gadolinium, fluid-attenuated inversion recovery (FLAIR), diffusion-weighted imaging (DWI), susceptibility-weighted imaging (SWI) and T2-weighted (T2w) sequences, Anatomical T1w MPRAGE pulse sequences were acquired for brain volumetric analysis.

Area of acquisition

Whole brain scans were used for further analysis

Diffusion MRI ☐ Used ☒ Not used

Preprocessing

Preprocessing software

The anatomical T1w images were automatically parcellated into 132 brain regions based on Neuromorphometrics atlas using the Neuromorphometrics toolbox. The atlasing methodology consists of two main steps. First, each image is segmented into three different brain tissue classes (CSF, gray matter, and white matter) using the "Segment" (unified segmentation) tool in SPM12 (Statistical Parametric Mapping Toolbox, v7771), which includes registration to the MNI (Montreal Neurological Institute) space. Second, the probabilistic atlas of each of the anatomical structures is spatially registered with the extracted gray and white matter tissue maps using the "Shoot" tool in SPM12, based on a nonlinear advanced registration algorithm. In all steps (segmentation, normalization) the default parameters were used.

Normalization

The T1w anatomical images were segmented into three different brain tissue classes (CSF, gray matter, and white matter) using the "Segment" (unified segmentation) tool in SPM12 (Statistical Parametric Mapping Toolbox, v7771), which includes registration to the MNI (Montreal Neurological Institute) space using the ICBM template. A nonlinear advanced registration algorithm was used with the probabilistic atlas of each of the anatomical structures to spatially registered with the extracted gray and white matter tissue maps using the "Shoot" tool in SPM12, and obtain the individual atlases.

Normalization template	The ICBM152 template was used in the brain tissue segmentation step.
Noise and artifact removal	N/A
Volume censoring	N/A

Statistical modeling & inference

Model type and settings	For volumetric brain imaging analysis, we checked the normal distributions of all variables using Shapiro-Wilk tests and visual inspection of the histograms. To test the equality of variances, Levene's test was applied. Clinical and demographic variables were compared between groups with Independent t-test, Mann-Whitney-U test, or Chi-square tests where appropriate. Regional volumes were compared between groups using a linear regression model. The additional covariates were age, sex, age*sex interaction, MRI magnetic field strength, and Total Intracraneal Volume (TIV). Choroid plexus volume (CPV) was adjusted for TIV and was compared by Mann-Whitney-U test. We checked whether the dependent variable's variance is equal between the groups by performing Levene's test of equal variances. The p-values were adjusted for multiple comparisons using FDR. The associations between brain regional volume and clinical measures were assessed using partial correlation. The method allows calculating the linear partial correlation between our variables of interest adjusting for different covariates. Our covariates were: age, sex, age*sex interaction, MRI magnetic field strength, and TIV. We adjusted for multiple comparisons using an FDR method.
Effect(s) tested	Main effect of group in the linear regression model.

Specify type of analysis: ☐ Whole brain ☒ ROI-based ☐ Both

Anatomical location(s)	The anatomical T1w images were automatically parcellated into 132 brain regions based on Neuromorphometrics atlas using the Neuromorphometrics toolbox. The atlasing methodology consists of two main steps. First, each image is segmented into three different brain tissue classes (CSF, gray matter, and white matter) using the "Segment" (unified segmentation) tool in SPM12 (Statistical Parametric Mapping Toolbox, v7771), which includes registration to the MNI (Montreal Neurological Institute) space. Second, the probabilistic atlas of each of the anatomical structures is spatially registered with the extracted gray and white matter tissue maps using the "Shoot" tool in SPM12, based on a nonlinear advanced registration algorithm. In all steps (segmentation, normalization) the default parameters were used.

Statistic type for inference (See Eklund et al. 2016)	Region-wise statistics.
Correction	False discovery Rate (FDR) for multiple comparisons correction

Models & analysis

n/a	Involved in the study
☒	☐ Functional and/or effective connectivity
☒	☐ Graph analysis
☒	☐ Multivariate modeling or predictive analysis
